# Genetic Encoding and Enzymatic Deprotection of a Latent Thiol Side Chain to Enable New Protein Bioconjugation Applications

**DOI:** 10.1002/anie.202102343

**Published:** 2021-06-11

**Authors:** Marie Reille‐Seroussi, Pascal Meyer‐Ahrens, Annika Aust, Anna‐Lena Feldberg, Henning D. Mootz

**Affiliations:** ^1^ Institute of Biochemistry University of Münster Corrensstraße 36 48149 Münster Germany

**Keywords:** genetic code expansion, nanobody, penicillin G acylase, regioselective labeling, thiol bioconjugation

## Abstract

The thiol group of the cysteine side chain is arguably the most versatile chemical handle in proteins. To expand the scope of established and commercially available thiol bioconjugation reagents, we genetically encoded a second such functional moiety in form of a latent thiol group that can be unmasked under mild physiological conditions. Phenylacetamidomethyl (Phacm) protected homocysteine (HcP) was incorporated and its latent thiol group unmasked on purified proteins using penicillin G acylase (PGA). The enzymatic deprotection depends on steric accessibility, but can occur efficiently within minutes on exposed positions in flexible sequences. The freshly liberated thiol group does not require treatment with reducing agents. We demonstrate the potential of this approach for protein modification with conceptually new schemes for regioselective dual labeling, thiol bioconjugation in presence of a preserved disulfide bond and formation of a novel intramolecular thioether crosslink.

## Graphical abstract

Enzymatic deprotection of a latent thiol under mild and physiological conditions expands the scope of classic thiol bioconjugation. New reaction schemes enable regioselective labeling and dual modifications for a variety of preparative applications and using the established and commercially available plethora of thiol‐directed reagents.

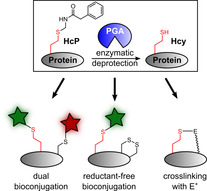

## Introduction

The cysteine thiol group has several unique properties that establish its outstanding role in the set of the ubiquitous 20 proteinogenic amino acids.^[1]^ For example, it serves as catalytic residue in various enzymes, it forms a covalent disulfide linkage in protein folding, it can be found both in the hydrophobic core and exposed to the solvent owing to its amphipathic character, and it is the target for many posttranslational modifications. The underlying unique nucleophilic and redox properties are also the basis for the importance of cysteine in chemical bioconjugation^[2]^ and ligation reactions^[3]^ for protein modification and synthesis. Only the rare selenol group of selenocysteine is of similar versatility.

To exploit the unique reactivities beyond the opportunities offered by a native or artificially introduced cysteine, several thiol‐ and selenol‐containing unnatural amino acids have been genetically incorporated in either free^[4]^ or protected form.^[5]^ The even higher reactivity of the selenocysteine side chain allows for chemoselective bioconjugation in the presence of thiol groups.^[6]^ However, a free selenol group can undergo undesired oxidation to diselenide and mixed sulfide‐selenide bonds during protein biosynthesis, folding and purification. In order to more generally enable a selective functionalization of an additional thiol or selenol group, their incorporation in a protected form is highly desirable. To date, all of the reported cases of such latent thiols or selenols employed photo‐chemical or chemical deprotection schemes that require conditions potentially damaging to the protein of interest (POI). For example, UV irradiation to deprotect photocaged cysteine, selenocysteine and homocysteine side chains can be harmful to disulfides in the protein and transforms the liberated thiol group into a reactive radical species.^[7]^ Photocage groups can also suffer from premature release or cellular conversion into inactive forms.^[8]^ Chemical deprotection of the thiazolidine‐protected cysteine moiety in thiaprolyl‐lysine^[5d]^ and the allyl‐protected selenocysteine analog^[5e]^ require high concentrations of methoxyamine at low pH and a palladium catalyst, respectively, that will be problematic for many proteins.

Thus, the genetic incorporation into proteins of a latent thiol or selenol group, that can be efficiently deprotected under native, mild and non‐destructive conditions, has not been reported so far. We envisioned that such a building block would represent a formidable addition to the protein chemist's toolbox to enable novel chemical manipulation schemes with chemo‐ and regioselectivity in presence of native cysteines and disulfides, and that can be performed under mild conditions.

**Scheme 1:**
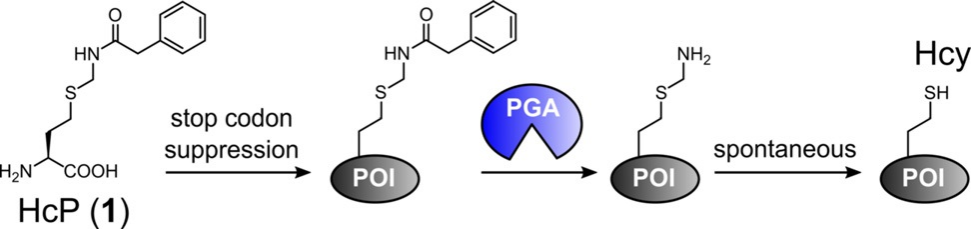
Concept of HcP incorporation and enzymatic deprotection. POI=protein of interest.

To address this challenge, we aimed to develop an enzymatic deprotection scheme to liberate a latent thiol in a novel non‐canonical amino acid that is incorporated into proteins by the genetic code expansion technology.^[9]^ Here we report on the incorporation of phenylacetamidomethyl (Phacm) protected homocysteine (HcP; **1**) and its enzymatic deprotection to homocysteine (Hcy) under physiological conditions using penicillin G acylase (PGA; Scheme 1). HcP could be efficiently deprotected within minutes using catalytic amounts of PGA, but depending on structural accessibility in the protein. We demonstrate the utility of this latent thiol group with a set of unique new applications based on thiol chemistry, ranging from selective dual labeling and selective single labeling under preservation of disulfide bonds to a novel intramolecular crosslinking approach.

## Results and Discussion

### Genetic Incorporation and Enzymatic Deprotection of Hcy(Phacm) (HcP)

We chose the Phacm group for protecting a latent thiol because PGA is known to recognize and cleave its phenylacetyl (Pac) portion and the liberated thioaminal would further decompose to reveal the Hcy core structure (Scheme 1). The heterodimeric PGA enzyme is used on an industrial scale to remove the Pac group from penicillin G; a key step in the production of semi‐synthetic penicillin variants.^[10]^ PGA does not cleave regular peptide bonds.[^10^, ^11^] It is also utilized on short polypeptide substrates in solid phase peptide synthesis (SPPS) to remove these groups from orthogonally protected Lys(Pac) and Cys(Phacm).[^11^, ^12^]

**Figure 1:**
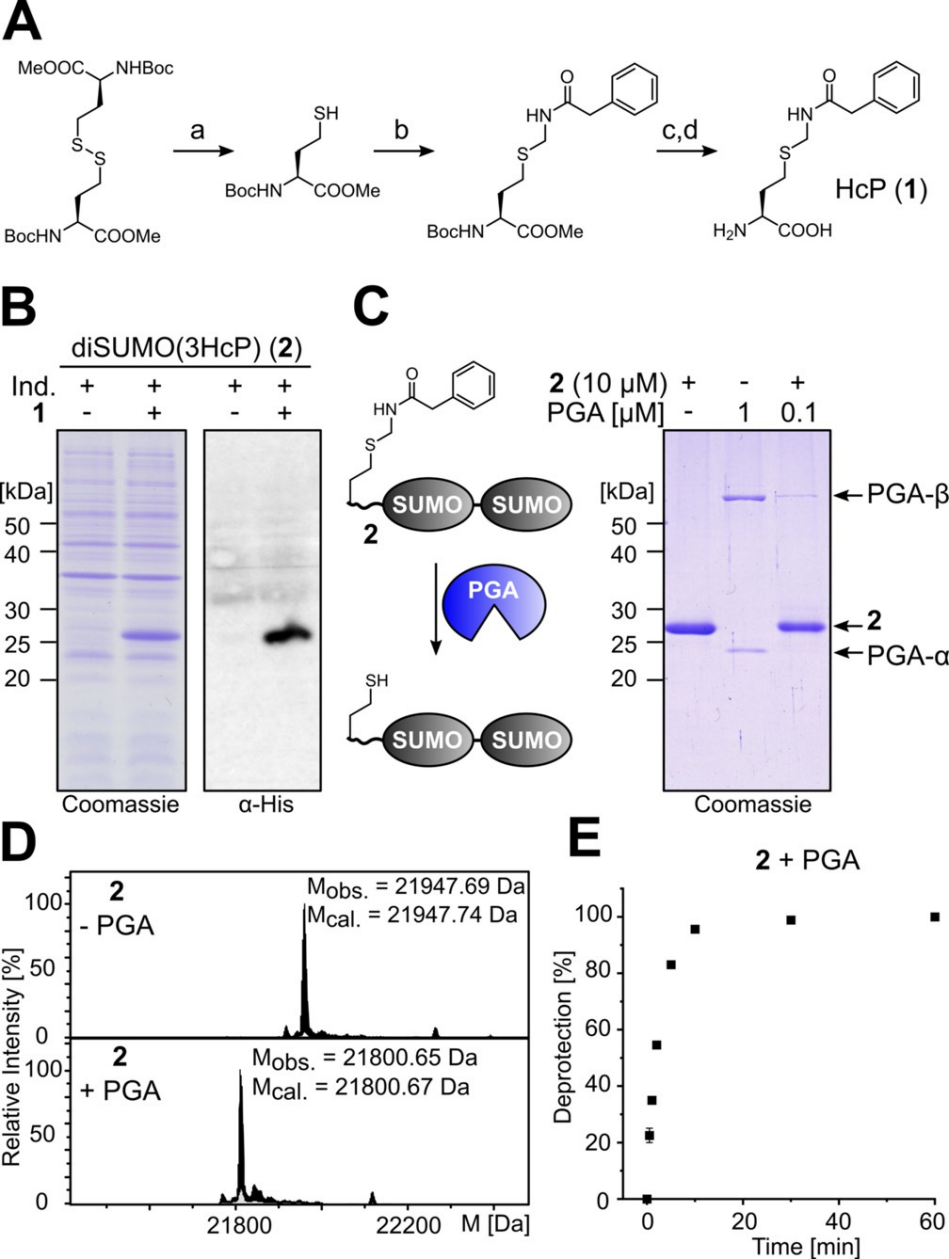
Synthesis, incorporation and deprotection of HcP (**1**). A) Scheme of synthesis. *a*=1.1 equiv. tri‐*n*‐butylphosphine in DCM/H_2_O; *b*=1.5 equiv. *N*‐hydroxymethylphenylacetamide, 0.08 equiv. TsOH in dioxane; c=LiOH in H_2_O/dioxane; *d*=1.2 equiv. HSiEt_3_ in TFA/DCM. Yield=46 %. B) Coomassie‐stained and α‐His western blot SDS‐PAGE analysis of incorporation of **1**. Ind.=induced expression. C) PGA‐His_6_‐mediated deprotection of HcP side chain in protein **2**. Left panel: Scheme of the reaction; right panel: Coomassie‐stained SDS‐PAGE of purified proteins and deprotection reaction as indicated. D) ESI‐MS analysis of the reaction shown in (C) with 0.01 equiv. PGA‐His_6_ (1 h, 25 °C). E) Time‐course of the analysis shown in C & D).

HcP (**1**) was synthesized according to the Scheme in Figure 1 A and obtained in good yield as a soluble TFA salt. To establish incorporation during protein translation by amber stop codon suppression we found that a mutated *Methanosarcina barkeri* pyrrolysine tRNA synthetase (PylRS*) evolved for lysine derivatives with bromoalkyl chains^[13]^ also accepted **1** as a substrate. *Escherichia coli* BL21(DE3) cells were transformed with two plasmids encoding the PylRS*/tRNA pair and a H_6_‐diSUMO (small ubiquitin related modifier) model protein (**2**) with an amber stop codon at the third position in front of the histidine tag, effectively representing the second position after removal of the start methionine. **1** was incorporated with good efficiency (yielding 13 mg L^−1^ expression culture of purified protein) when added at 2 mM to the growth medium (Figure 1 B). ESI‐MS analysis of the purified diSUMO(3HcP) protein (**2**) confirmed the expected mass with no unprotected species detectable. This result verified the incorporation of **1** and suggested that the Phacm group remained stable during expression and purification.

Having established incorporation of **1** via genetic code expansion, we investigated removal of the Phacm group from the model protein using PGA. We prepared purified PGA tagged with a hexahistidine sequence at the C terminus of the β subunit by recombinant expression in *E. coli* ^[14]^ and tested the enzyme at different concentrations. To our delight, incubation of the protein substrate **2** (10 μM) with just 0.01 equiv. PGA‐His_6_ led to quantitative deprotection, as confirmed by ESI‐MS, and was virtually complete already after 10 min (Figure 1 D & E). Even with a lower amount of PGA‐His_6_ (0.001 equiv.) we still observed complete deprotection after 2 h (Figure S1). No protein degradation could be detected, even at high PGA‐His_6_ concentrations (10 μM≙1 equiv., tested up to 24 h), consistent with previous reports that PGA does not cleave in the peptide backbone of proteins (Figure S2).^[11]^ The ESI‐MS analysis also allowed us to observe the thioaminal intermediate, further confirming the mechanism following PGA‐mediated removal of the Pac moiety (Figure S3). Of note, this and all subsequent MS analyses with other proteins showed no indication of a potential adduct formation with formaldehyde, suggesting that no such undesired effects are caused by this side product of the Phacm‐release mechanism (Scheme 1), likely due to the low concentration in the micromolar range.

### Structural Requirements for PGA‐Mediated HcP Deprotection

**Figure 2:**
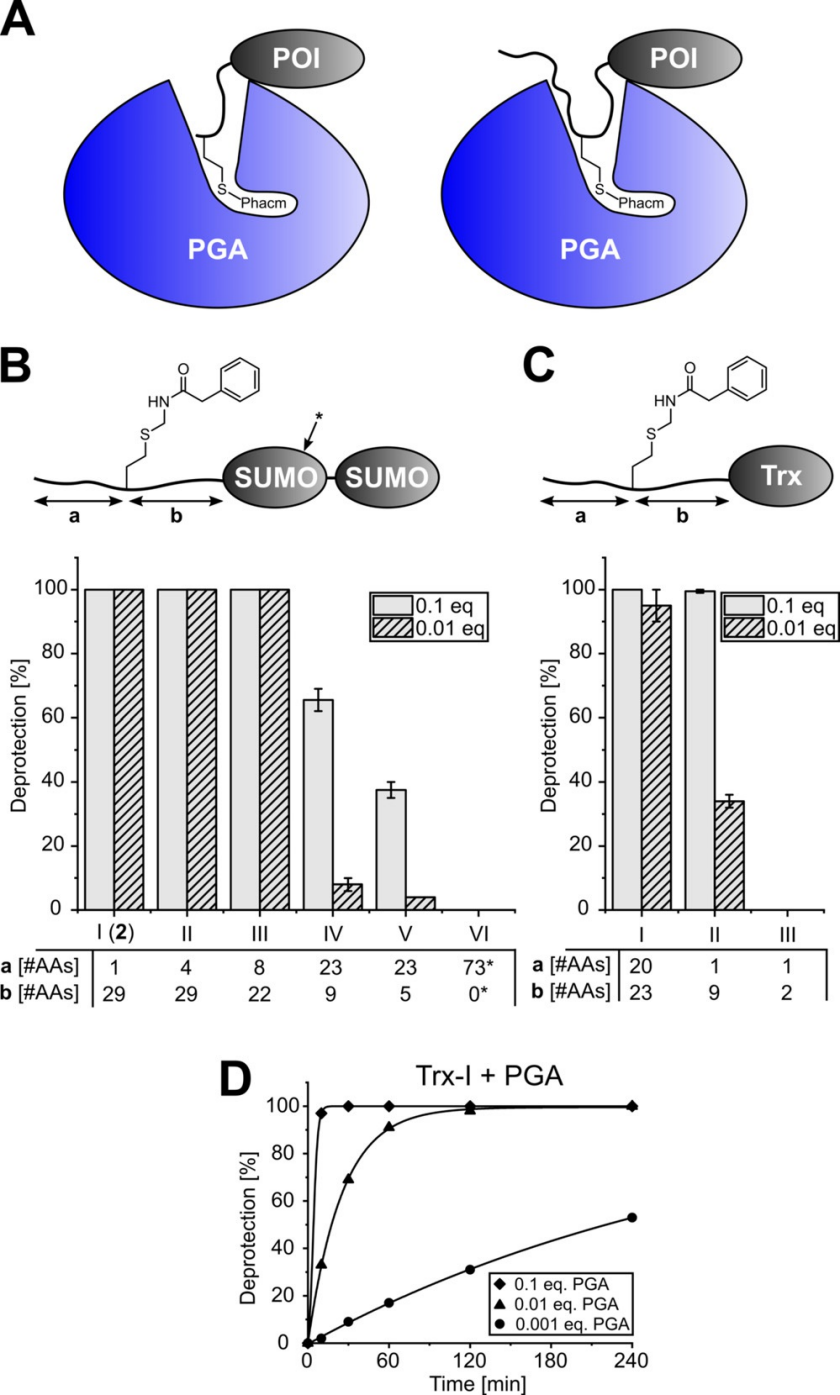
Deprotection efficiency of HcP (**1**) with PGA. A) One‐pass (left panel) and two‐pass model (right panel) for a flexible polypeptide chain to reach through the funnel into the active site of PGA. B) Deprotection yield of diSUMO constructs with **1** at varying positions (*t*=4 h). *=**1** on surface of globular part of SUMO. C) Deprotection yield of Trx constructs with **1** at varying positions (*t*=1 h). D) Deprotection time‐course of protein Trx‐I with different amounts of PGA‐His_6_.

We then studied the structural requirements for such efficient and rapid PGA‐mediated deprotection of HcP (**1**) in proteins. PGA's active site is at the bottom of a deep funnel that becomes increasingly constricted and should be easily accessible only by small molecules such as the native substrate penicillin G (Figure 2 A).^[15]^ Therefore, a clear dependency on the surrounding steric bulk was expected. The accessibility of the side chain of **1** should depend on the globular fold around its position.^[13a]^ In our initial deprotection assays (Figure 1), **1** was located close to the N terminus of an unstructured tail region, thus exhibiting nearly maximal accessibility. We systematically tested other positions with varying distance from the N terminus in diSUMO model proteins (Figures 2 B and S4–S6) and analyzed time‐dependent deprotection by ESI‐MS as described above. Constructs with **1** in a distance of 1, 4, or 8 aa from the N terminus and 20–29 aa away from the globular fold of the first SUMO domain were quantitatively deprotected under the chosen conditions (0.1 equiv. PGA‐His_6_, 4 h; see Figure 2 B). These three constructs were even quantitatively deprotected with 0.01 equiv. PGA‐His_6_ in ≤1 h (Figures 1 E, S3 and S6), suggesting that the position of **1** within an unfolded peptide stretch is largely irrelevant for a rapid and efficient deprotection as long as it is located with enough distance to structured regions.

We then tested deprotection of **1** on the surface of the first SUMO domain (substituting R61), representing the other extreme in terms of structural accessibility. The globular SUMO fold encompasses ≈72 aa. In fact, no deprotection could be observed, consistent with the notion that the SUMO domain would not fit deep enough into the funnel to the active site of PGA. Finally, we wondered about the minimally required distance of the **1** position relative to a global protein domain for efficient deprotection to occur. With **1** being only nine residues away from the globular SUMO domain, the efficiency was lowered to about 65 % (0.1 equiv. PGA‐His_6_, 4 h). A shorter distance of only 5 residues led to a further reduction of the efficiency (≈40 %; Figure 2 B).

Similar findings were obtained using *E. coli* thioredoxin (Trx; folded globular domain of ≈95 aa) with an artificial N‐terminal extension as another model substrate protein (Figures 2 C and S7). Notably, quantitative deprotection was observed with **1** being flanked by 20–23 aa on both sides as well as with **1** just 9 aa from the globular domain. In contrast, a distance of only 2 aa led to complete impairment (using 0.1 equiv. PGA‐His_6_ for 4 h). A further lowering of the PGA‐His_6_ concentration to 0.01 equiv. showed for both model proteins that positions with a distance of only 9 aa to the globular domain are deprotected less rapidly (Figure 2 B–D).

Together, these data are supporting a model that about ≥8–10 aa are needed in unstructured or stretched conformation to reach the PGA active site from the outer rim of its substrate funnel (of 25–30 Å distance) in a one‐pass arrangement as shown in Figure 2 A (left panel). Even a two‐pass arrangement of the polypeptide chain in the funnel is possible and can lead to efficient and rapid deprotection of **1**, suggesting a significant increase of possible protein substrates (Figure 2 B, right panel). HcP (**1**) in large globular structures and on flat surfaces is likely inaccessible for PGA but may still be cleaved at a slower rate depending on the architecture when applying longer incubation times and higher PGA amounts.^[13a]^

Of note, the rates and efficiencies observed here for PGA‐mediated deprotection reveal an unexpectedly high catalytic potential on protein substrates. They represent a dramatic improvement over previously reported PGA deprotection schemes of Lys(Pac) or Cys(Phacm) on both peptide[^12^, ^13^, ^16^] and protein[^13a^, ^17^] substrates, which were often incomplete and required overnight incubation. Next to the structural dependencies, it should be noted that PGA has typically been used as a commercial sample from industrial production batches, often in immobilized form. We recommend not to use such samples for protein applications due to potential problems with protein degradation and precipitation, likely due to their crude nature.^[13a]^

Having established protocols for HcP(**1**) incorporation and enzymatic deprotection to Hcy, we next aimed to develop new protocols in protein chemistry and protein labeling.

### Sequential and Regioselective Protein Dual‐Labeling using Bioconjugation of Thiol and Latent Thiol Groups

We reasoned that the latent thiol group of **1** would allow for a straight‐forward sequential and regioselective dual‐labeling strategy of a POI. Following labeling of a single Cys side chain, **1** would be converted to Hcy and the liberated thiol group then be labeled in a second reaction. Assuming no remaining reactive cysteine side chains after the first labeling step, this approach represents a quasi orthogonal labeling of two thiol groups. Importantly, both labeling steps of this protocol can employ well‐established and efficient thiol bioconjugation, which is appealing for its simplicity and availability of a plethora of commercial reagents. Other reported strategies for regioselective dual labeling involve two different functional moieties, including more specialized types for bioorthogonal conjugation reactions.^[18]^

**Figure 3:**
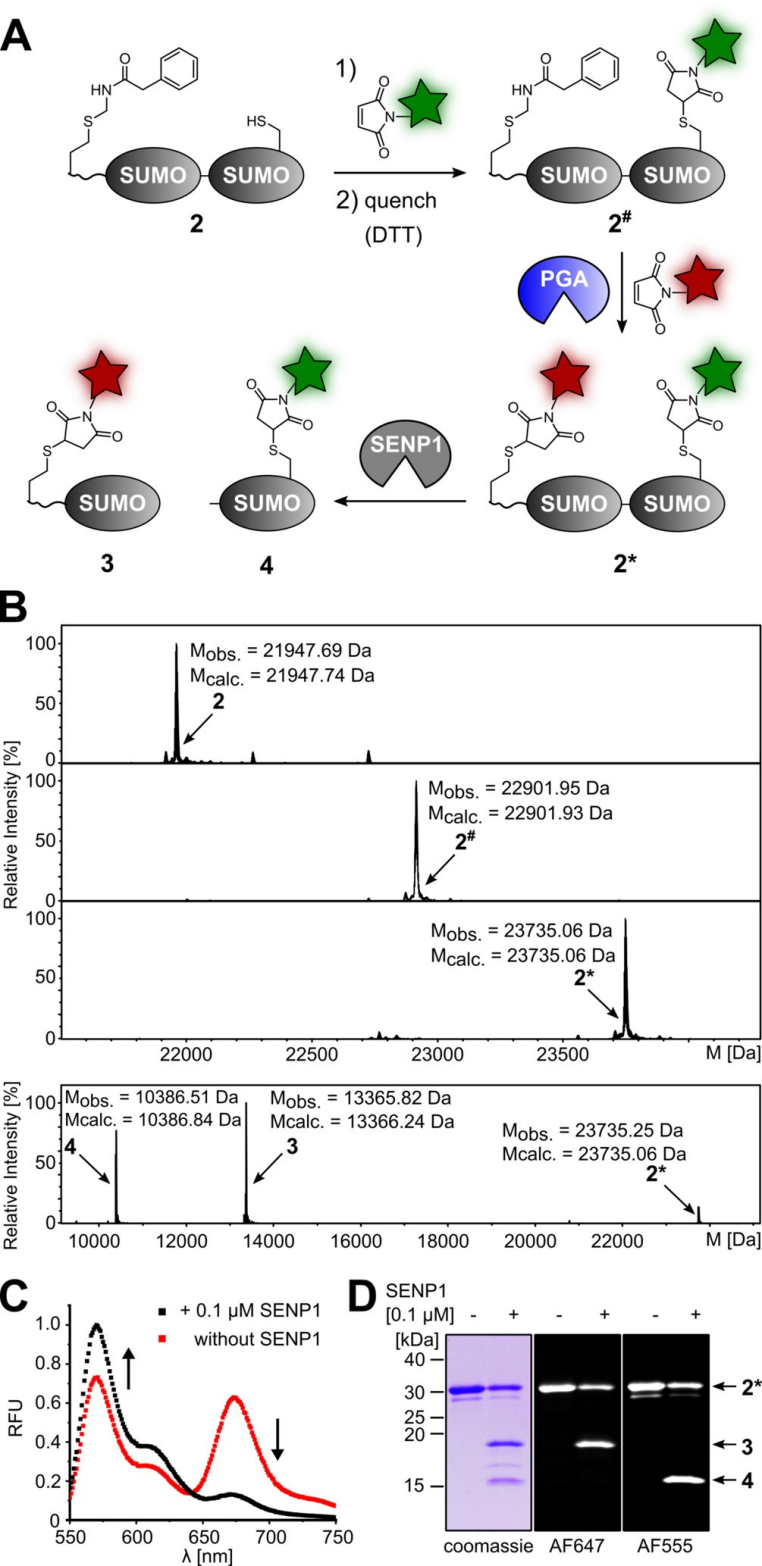
Regioselective Cys and Hcy dual labeling of proteins. A) Scheme to prepare diSUMO FRET sensor 2* and its proteolytic cleavage by SENP1. B) ESI‐MS analysis of the unpurified reaction mixtures to analyze intermediates and products. C) Fluorescence spectra of dually labeled 2* (2 μM) with and without treatment with SENP1 (0.1 μM, 2 h) using excitation at 520 nm. C) SDS‐PAGE analysis of the proteolytic cleavage reaction of 2* (10 μM) with SENP1 (0.1 μM, 2 h). Excitation of the gel was performed at 609 nm for AF647 and 535 nm for AF555.

Dual labeling of a protein with an acceptor and a donor dye is key to the Förster resonance energy transfer (FRET), a widely used technique to study intramolecular distances and conformational changes. We therefore tested the idea of sequential and regioselective Cys and Hcy labeling with a diSUMO FRET sensor. The design of the sensor was based on the above‐described model protein **2** with the latent thiol group of **1** in the N‐terminal region of the distal SUMO unit (Figure 3 A). This protein also contained a single cysteine (R61C) in the proximal SUMO unit. In the first bioconjugation step, the thiol group of the cysteine was labeled with the donor dye AlexaFluor 555 (AF555) as a maleimide reagent to give **2^#^** (Figure 3 A). Following quenching with DTT (100 equiv.) and subsequent removal of excess quencher and dye by dialysis, we added PGA‐SBP and AF647 maleimide as the acceptor dye to simultaneously trigger conversion of HcP to Hcy and labeling of the latter to give dually labeled 2* (Figure 3 A). PGA‐SBP carries a streptavidin‐binding peptide instead of the His_6_ tag and therefore could easily be removed together with the excess AF647 dye by Ni‐NTA purification of the His_6_‐tagged 2*. Notably, PGA and PGA‐SBP have no cysteines and therefore do not cause any background labeling in such one‐pot reactions. The dual labeling of 2* without acceptor‐acceptor or donor‐donor conjugates was confirmed by ESI‐MS (Figure 3 B). 2* exhibited intramolecular FRET (Figure 3 C), which was lost by enzymatic cleavage into the AF647 and AF555‐labeled SUMO monomers **3** and **4**, respectively, with the SUMO protease SENP1 (Figure 3 B bottom panel, Figures 3 C & D and S8).^[18e]^ Together, these data confirmed the intended regioselective conjugation of each SUMO monomer with just one specific dye.

**Figure 4:**
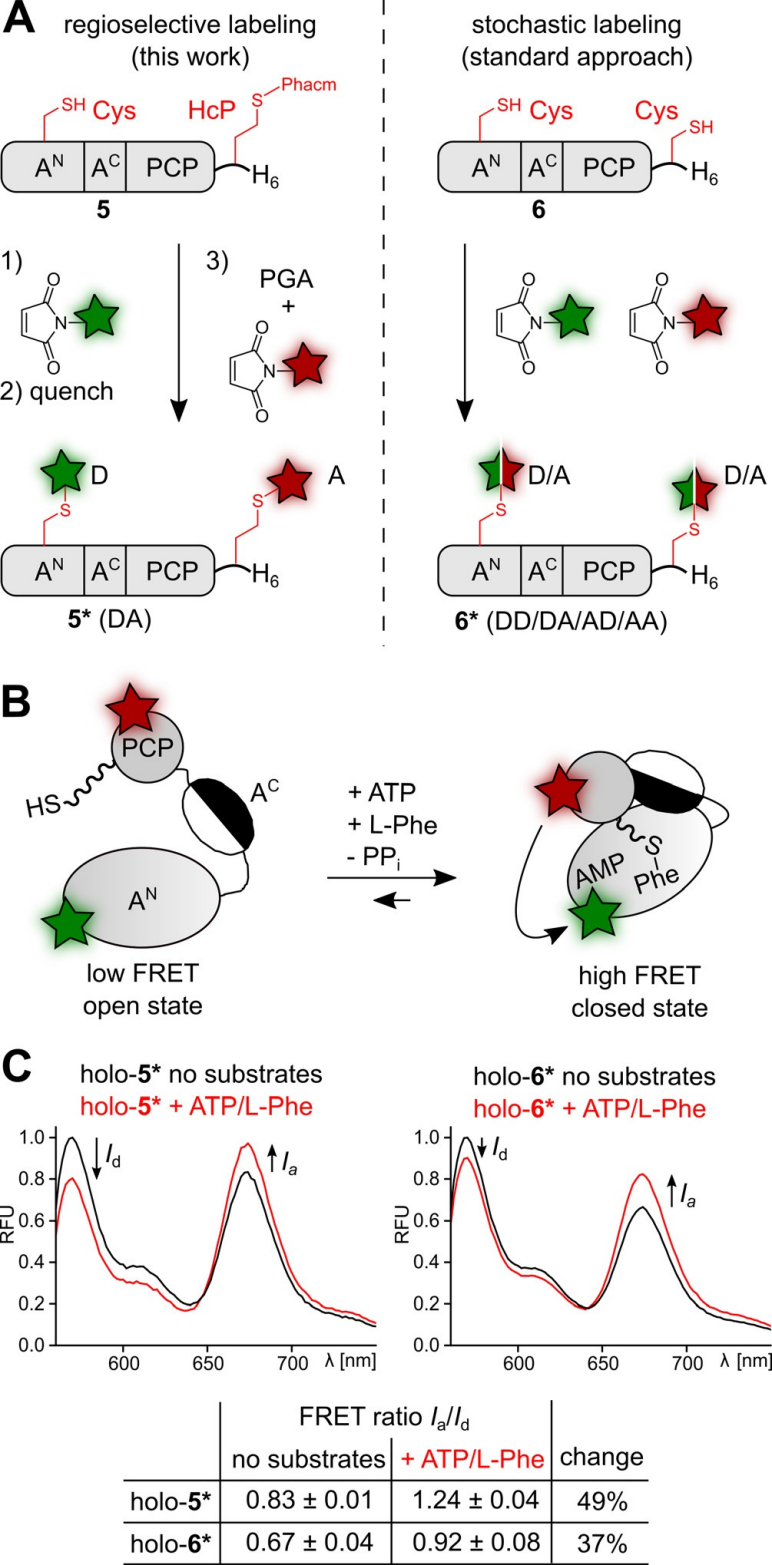
Intramolecular multidomain NRPS FRET‐sensor improved by regioselective dual labeling. A) Schematic comparison of regioselective and stochastic labeling. B) Scheme of conformational change of the NRPS di‐domain A‐PCP‐FRET sensor following substrate addition. C) Fluorescence spectra of holo‐5* or holo‐6* (0.3 μM) to monitor FRET ratios with and without substrates (using excitation at 520 nm). Measurements with substrates were performed 30 min after adding 2 mM ATP and 2 mM L‐Phe.

We then aimed to demonstrate the advantage of the new regioselective dual‐labeling protocol over the typically used stochastic labeling of two cysteines in terms of the FRET response. Whereas the stochastic labeling cannot avoid the partial dual labeling with only acceptor and only donor dyes (Figure 4 A, right panel), no such unproductive species are formed in our new approach and hence an increase in dynamic FRET range would be expected (Figure 4 A, left panel). To this end, we designed a new nonribosomal peptide synthetase (NRPS) FRET sensor to monitor the interaction of its adenylation (A) and peptidyl‐carrier‐protein (PCP) domains, which together comprise 71 kDa. The in‐solution analysis of the domain interaction is important to understand the timing and dynamics of conformational changes in these multi‐domain biosynthetic enzymes.^[19]^ The only currently reported FRET sensor design for these large, multidomain proteins utilized EGFP as a large fluorescent protein donor dye, which is not optimal due to its possible impact on domain mobility.^[19]^ For a design based on two small synthetic dyes, we introduced a single cysteine at position N152C by site‐directed mutagenesis in the large A^N^ subunit of the A domain^[19a]^ and fused a short tag (AGV(HcP)TEH_6_) containing the new amino acid at the C‐terminal end of the PCP domain to give A‐PCP(N152C/HcP‐tag) (construct **5**). For comparison, cysteine was used at both positions to give the control construct A‐PCP(N152C/Cys‐tag) (**6**) (Figure 4 A). The Cys‐HcP construct **5** was labeled with AF555 and AF647 maleimides according to our new sequential protocol involving PGA‐SBP to deprotect the latent thiol group. Excess dye and PGA‐SBP were separated off by Ni‐NTA chromatography. Successful regioselective dual labeling (91 %) was confirmed by SDS‐PAGE and ESI‐MS (Figures S9A and S10). The Cys‐Cys control construct **6** was bioconjugated under the same conditions, however, by adding AF555 and AF647 maleimides simultaneously as a mixture to afford stochastic labeling of the two cysteines (Figure S9B). Subsequently, both labeled proteins 5* and 6* were converted into the catalytically active holo‐forms by addition of the 4′‐phosphopantetheinyl (Ppant) transferase Sfp^[20]^ and coenzyme A (Figure S10) to give the FRET sensors holo‐5* and holo‐6*.

The A‐PCP FRET sensors were then biochemically and biophysically characterized. The enzyme catalyzes L‐Phe‐AMP formation from the substrates L‐Phe and ATP as well as the subsequent covalent binding of the amino acid as an L‐Phe‐thioester on the Ppant prosthetic group. These chemical reactions correlate with a shift of the conformational equilibrium to a preferential binding of the PCP domain to the A domain (Figure 4 B).^[19]^ Importantly, in the absence of substrates, the regioselectively labeled sensor holo‐5* showed a higher FRET ratio (acceptor intensity over donor intensity=*I*_a_/*I*_d_) compared to holo‐6*, consistent with the lack of the unproductive donor‐donor and acceptor‐acceptor combinations (FRET ratios of 0.83 vs. 0.67, respectively; Figure 4 C). Both FRET sensors reported on the conformational change induced by the addition of substrates ATP and L‐Phe. Again, the regioselective design of holo‐5* proved to be more sensitive as it led to a greater change in the FRET ratio (49 % vs. 37 % for holo‐6*, respectively; Figure 4 C), corresponding to a higher dynamic range. Together, these findings clearly demonstrate the advantage of regioselective over stochastic labeling in FRET studies, and show the utility of our new labeling protocol for such purposes.

### Latent Thiol Bioconjugation in Presence of Intact Cysteine Disulfide

In another novel strategy for protein modification we aimed to take advantage of the fact that the latent thiol group of **1** does not interfere with existing disulfide bonds in the protein during expression and purification. This point is particularly noteworthy in conjunction with the nascent nature of the Hcy thiol group following deprotection by PGA, which should allow quantitative bioconjugation without a preceding treatment with reductants like TCEP or DTT, as is typically required for cysteine bioconjugation following protein expression and purification. We hypothesized that protein thiol bioconjugation on the latent thiol group of **1** should be possible with preservation of disulfide bonds and with avoiding otherwise potentially harmful reducing conditions.

**Figure 5:**
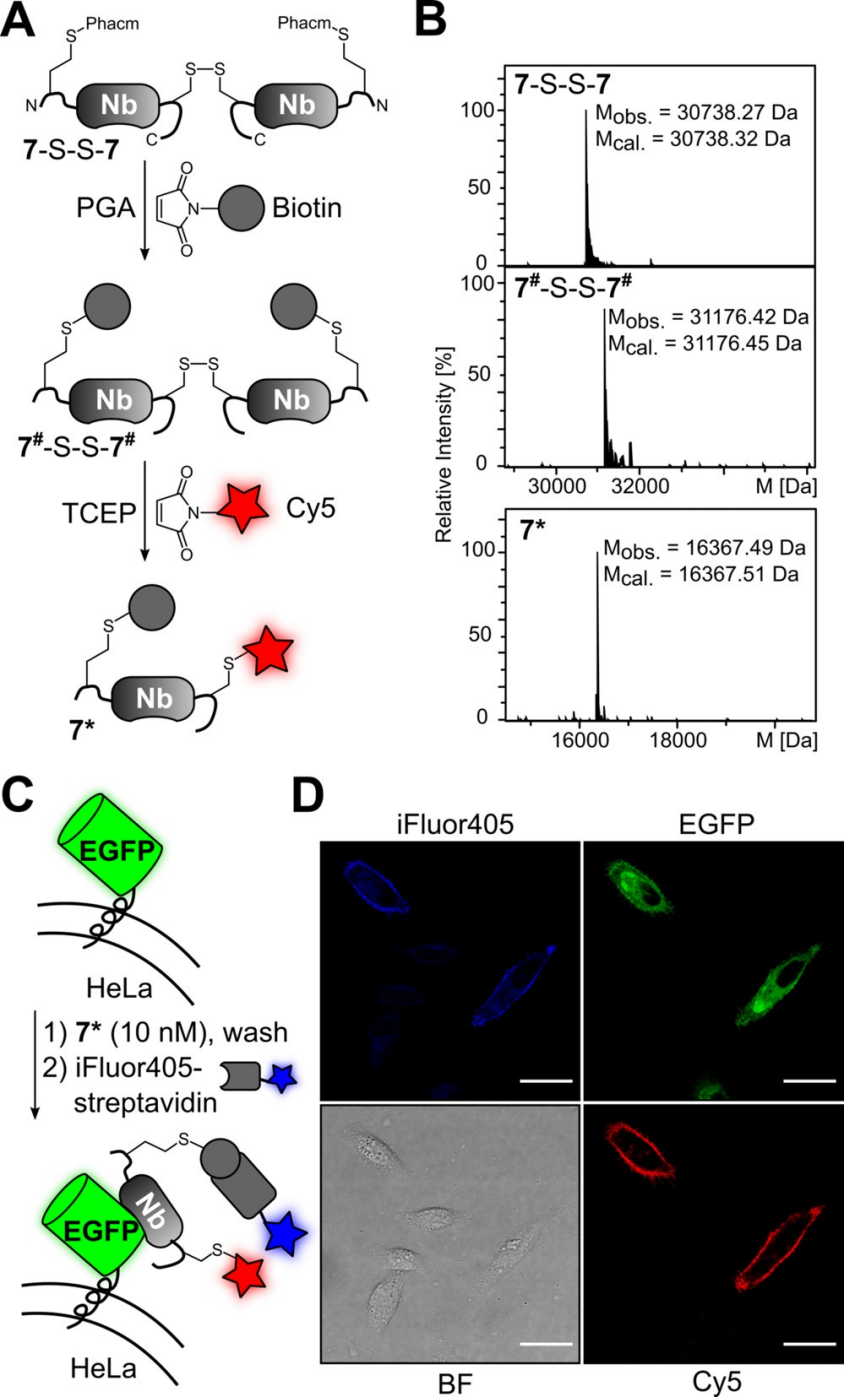
Latent thiol bioconjugation in presence of disulfide bond. A) Reaction Scheme involving a disulfide‐linked anti‐GFP nanobody (Nb) with subsequent dual labeling after reducing the disulfide bond. B) ESI‐MS analysis of unpurified intermediates in the reaction Scheme shown in (A). C) Scheme of Nb‐mediated cell surface labeling of HeLa cells expressing EGFP. D) Confocal microscopy images of transiently transfected HeLa cells to present EGFP as shown in (C). Note that non‐transfected cells did not bind the nanobody. Scale bar=25 μm.

Nanobodies (Nbs) are single‐domain antibody fragments derived from heavy‐chain‐only antibodies with countless applications in protein biochemistry, diagnostics and therapy.^[21]^ We noticed that when nanobodies are expressed into the periplasm of *E. coli*, an extra single cysteine in a short appended tag sequence tends to form a disulfide with a second monomer to give a covalent homodimer. We therefore aimed to test our hypothesis by selectively bioconjugating the latent thiol group of **1** in the presence of this disulfide bond between the two nanobodies. In an additional extension, the disulfide could be reduced to give the monomeric nanobody and the unpaired cysteine be used for a second labeling reaction (Figure 5 A).

To this end, we prepared a GFP‐enhancer nanobody^[22]^ with a C‐terminally appended cysteine and additionally fused a short N‐terminal tag to introduce the latent thiol group of **1**. The resulting protein **7** was purified as a disulfide‐bridged dimer **7**‐S‐S‐**7** (Figure 5 B) without any detectable free thiol of a monomeric species (Figure S11). Addition of PGA‐His_6_ (0.02 equiv., 1 h) to convert **1** into Hcy and in situ labeling with biotin maleimide afforded the desired conjugate **7^#^**‐S‐S‐**7^#^** with the intact disulfide bond in quantitative yield, as confirmed by ESI‐MS (Figure 5 B, top panels). Furthermore, to achieve subsequent dual labeling, TCEP was added to **7^#^**‐S‐S‐**7^#^** to reduce the disulfide and give the monomeric species of **7^#^**. Subsequent addition of Cy5 maleimide led to bioconjugation of the unpaired cysteine side chain and furnished the dually labeled nanobody 7* (Figure 5 B, bottom panel and Figure S12; note that under these conditions the other, internal disulfide bond of the nanobody remained unaffected). Finally, we proved the functional integrity of 7* (10 nM) by its specific binding to EGFP presented on HeLa cells^[23]^ and by visualizing the biotin moiety using iFluor405 conjugated streptavidin (Figure 5 C and D).

### A Latent Thiol for Enzyme‐Activated Protein Crosslinking

Finally, the latent thiol group of **1** inspired us to a novel strategy to create stable protein crosslinks. We reasoned that as long as the latent thiol group is kept protected, the thiol group of a free cysteine could be chemically converted into a suitable electrophile. Deprotection of the latent thiol by PGA then reveals the nucleophilic reaction partner.

**Figure 6:**
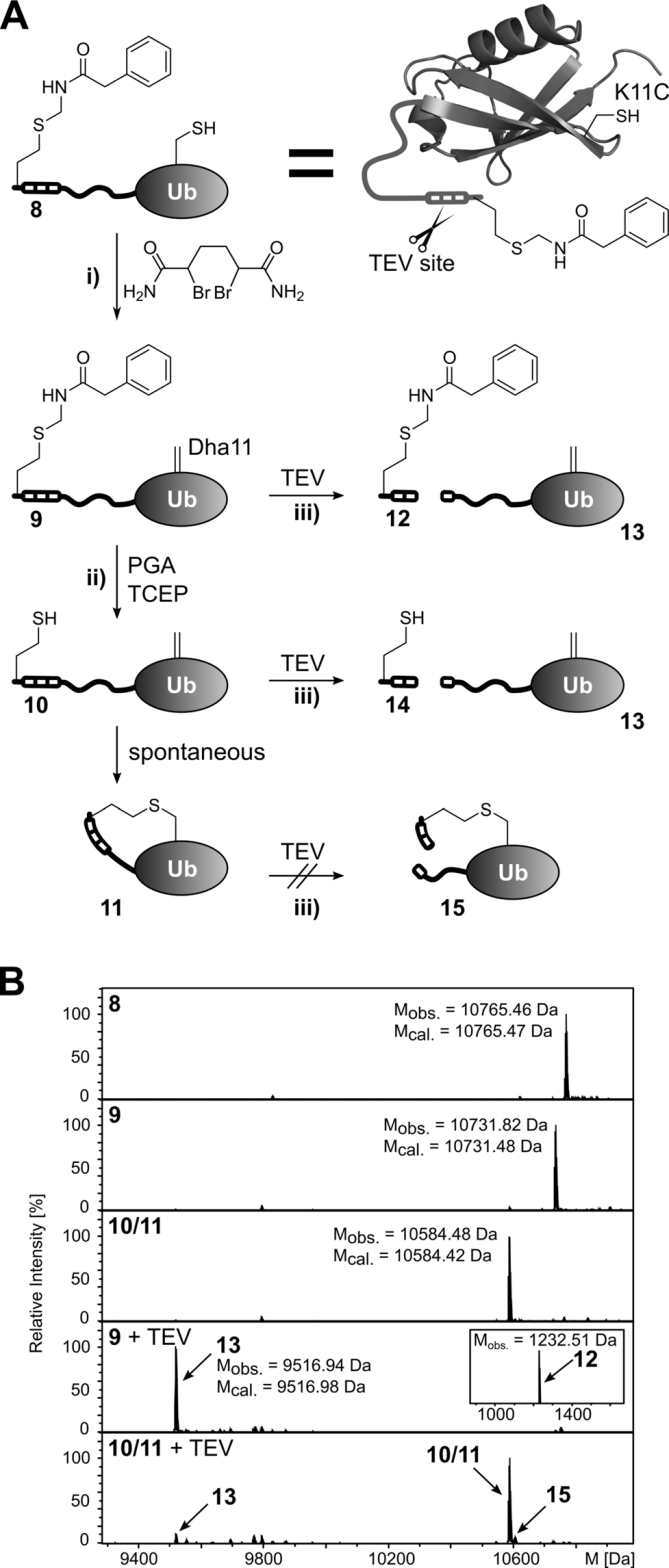
Intramolecular protein crosslinking using the latent thiol group. A) Scheme of the strategy involving the conversion of a single cysteine into an electrophile. The structural representation of the Ub model protein is based on pdb‐file 1UBQ. Reaction conditions: i) dibromoadipic amide (10 equiv., overnight, 37 °C); ii) PGA‐His_6_ (0.01 equiv.), TCEP (5 equiv.); overnight); iii) TEV protease (0.1 equiv., 3 h). B) ESI‐MS analysis of the reaction intermediates and products (**12**: M_cal._=1232.52 Da).

To test this idea, we chose to create an intramolecular thioether covalent crosslink on ubiquitin as the model protein (Figure 6 A). We incorporated **1** into an unstructured N‐terminal peptide extension, that also contained a TEV protease cleavage site, and introduced a single cysteine by site‐directed mutagenesis on the surface of Ub's globular fold (K11C) to give protein **8**. The correct masses of this protein and the following modification steps were confirmed by ESI‐MS (Figure 6 B). Treatment of purified **8** with dibromoadipic amide converted Cys11 into dehydroalanine (Dha11; protein **9**).^[24]^ Following removal of excess 2,5‐dibromoadipic amide we added PGA‐His_6_ (0.01 equiv.) to quantitatively deprotect the latent thiol of **1** to Hcy (protein **10**). Overnight incubation was necessary to allow for the slow spontaneous addition of the Hcy thiol to the Dha double bond to give cyclized protein **11** with the novel protein crosslink. However, since this reaction does not change the molecular mass of the protein we were unable to distinguish **11** from **10** by ESI‐MS. To verify that the cyclization reaction had occurred, we reasoned that the constrained conformation of the cyclized peptide chain in **11** would likely twist the TEV cleavage site from its stretched conformation necessary for recognition by TEV protease and thereby partially or completely block TEV cleavage of **11** into **15**. In contrast, the uncyclized structures in **9** and **10** should be quantitatively cleaved (Figure 6 A; right panels). Indeed, we found **9** to be quantitatively cleaved by TEV protease into **12** and **13**. In contrast, following incubation of the potential mixture of uncyclized **10** and cyclized **11** with TEV we could only detect a weak signal for cleavage product **13** and most of the signal for **10**/**11** remained unchanged (Figure 6 B, bottom panel). This finding is consistent with our hypothesis that the cyclized protein **11** was resistant against TEV cleavage, and suggests that a near quantitative intramolecular crosslink to **11** had occurred. Analysis of the protein species by SDS‐PAGE electrophoretically separated cleaved **13** from **10**/**11** and corroborated an efficient yield of >90 % for the crosslink reaction (Figure S13).

Together, these results show that the latent thiol group in **1** can be used to efficiently introduce novel crosslinks into proteins. Notably, the formed thioether crosslink is of minimal atom economy, also due to the small size of the Hcy side chain. The latter contrasts to the mostly long and bulky side chains incorporated by the genetic code expansion technology for related purposes.^[25]^

## Conclusion

We have reported the genetic encoding of the novel non‐canonical amino acid HcP (**1**) with a latent thiol group and its enzymatic deprotection to Hcy. The PGA‐mediated deprotection was found to be surprisingly rapid and efficient at sterically well‐accessible positions in unstructured regions, but was poor or impossible on flat surfaces of stably folded globular proteins. Importantly, the deprotection is performed under mild, physiological, non‐denaturing and non‐destructive conditions to the protein of interest, representing a significant advancement in terms of preparative utility over previously reported chemical or photochemical deprotection schemes of latent thiol or selenol groups. We have demonstrated the potential of the latent thiol group in several novel approaches of selective chemical modification of proteins, thereby significantly expanding the scope of thiol bioconjugation. The variety of these approaches reflect the versatility of thiol group chemistry. Protein thiol bioconjugation is a classical and well‐established technique, and is still highly attractive due to its simplicity, chemoselectivity, efficiency and wide‐spread use. The synthesis of the required unnatural amino acid **1** is easy to perform. The requirement for bioorthogonal reactions is circumvented in our chemical modification schemes. Bioorthogonal reactions require more sophisticated reagents of more restricted availability, in particular for dual labeling,^[18]^ and are of more restricted applicability in many regards, for example as caused by the interference of cyclooctynes with free cysteines.^[26]^ Our reported examples include a) two different routes to regioselective and consecutive dual labeling of cysteine and homocysteine (Hcy) as two quasi‐orthogonal thiol groups, b) selective thiol conjugation under preservation of disulfide bonds that are otherwise sensitive to reducing conditions, and c) a protein crosslinking strategy to introduce stable thioether bridges. These protocols would not have been possible without the latent thiol group. We believe that they present powerful new tools for the protein chemist and biochemist and that the novel latent thiol group will enable many other new and unique applications.

## Conflict of interest

The authors declare no conflict of interest.

## Supporting information

As a service to our authors and readers, this journal provides supporting information supplied by the authors. Such materials are peer reviewed and may be re‐organized for online delivery, but are not copy‐edited or typeset. Technical support issues arising from supporting information (other than missing files) should be addressed to the authors.

Supplementary
