# Supplementary material for: Genetic Encoding and Enzymatic Deprotection of a Latent Thiol Side Chain to Enable New Protein Bioconjugation Applications
Source: Angew Chem Int Ed Engl. 2021 Jun 11;60(29):15972–9. doi: 10.1002/anie.202102343 (PMC8361980; doi:10.1002/anie.202102343)

Angewandte Chemie
Eine Zeitschrift der Gesellschaft Deutscher Chemiker

Supporting Information

Genetic Encoding and Enzymatic Deprotection of a Latent Thiol Side Chain to Enable New Protein Bioconjugation Applications

*Marie Reille-Seroussi[+], Pascal Meyer-Ahrens[+], Annika Aust[+], Anna-Lena Feldberg, and Henning D. Mootz**

anie_202102343_sm_miscellaneous_information.pdf

Table of Contents

Supporting Figures	page S3
Chemical Synthesis	page S14
Biochemical Methods	page S16
Supporting Table	page S20
Supporting References	page S22
Appendix	page S23

Supporting Figures

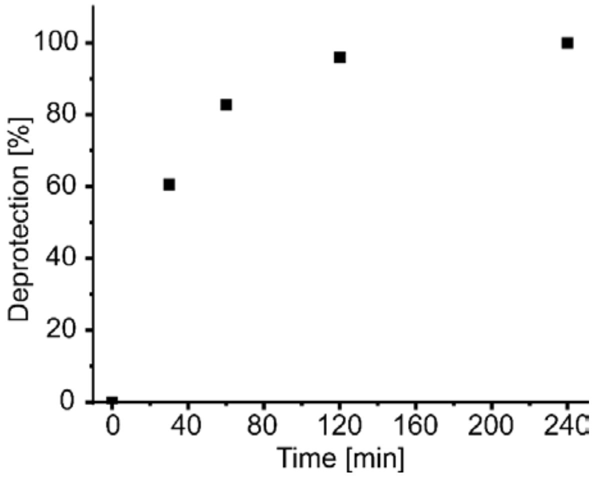

Figure S1: Time-dependent HcP deprotection. DiSUMO-I (**2**) (10 µM) was incubated with 0.001 eq. PGA-His6 and the reaction monitored by ESI-MS.

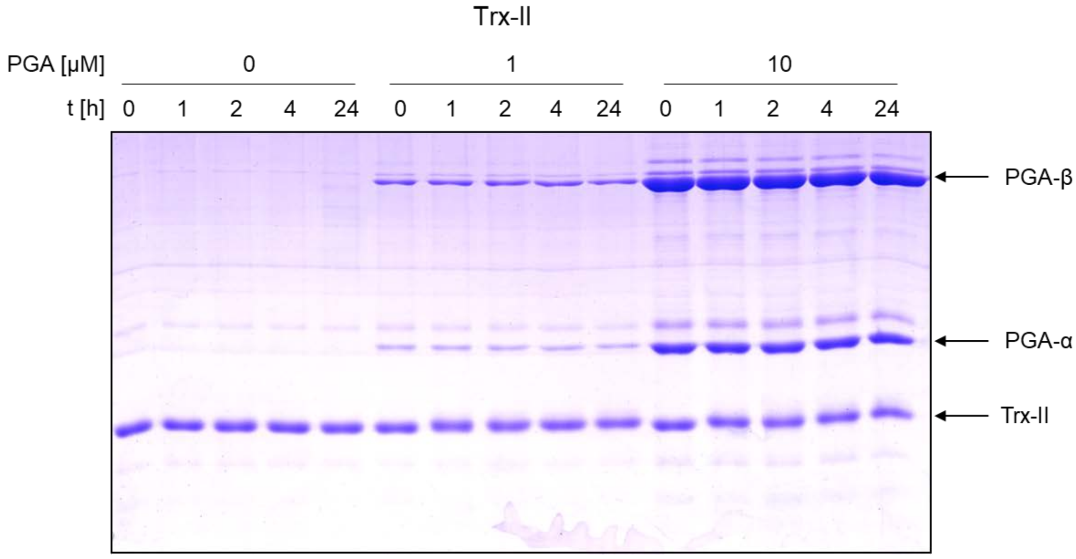

Figure S2: Protein backbone stability towards PGA. Trx-II (10 µM) was incubated with PGA-His6 at different concentrations for the indicated periods of time. SDS-PAGE gel analysis showed no observable protein degradation by PGA. Note that some unassigned protein bands represent protein impurities.

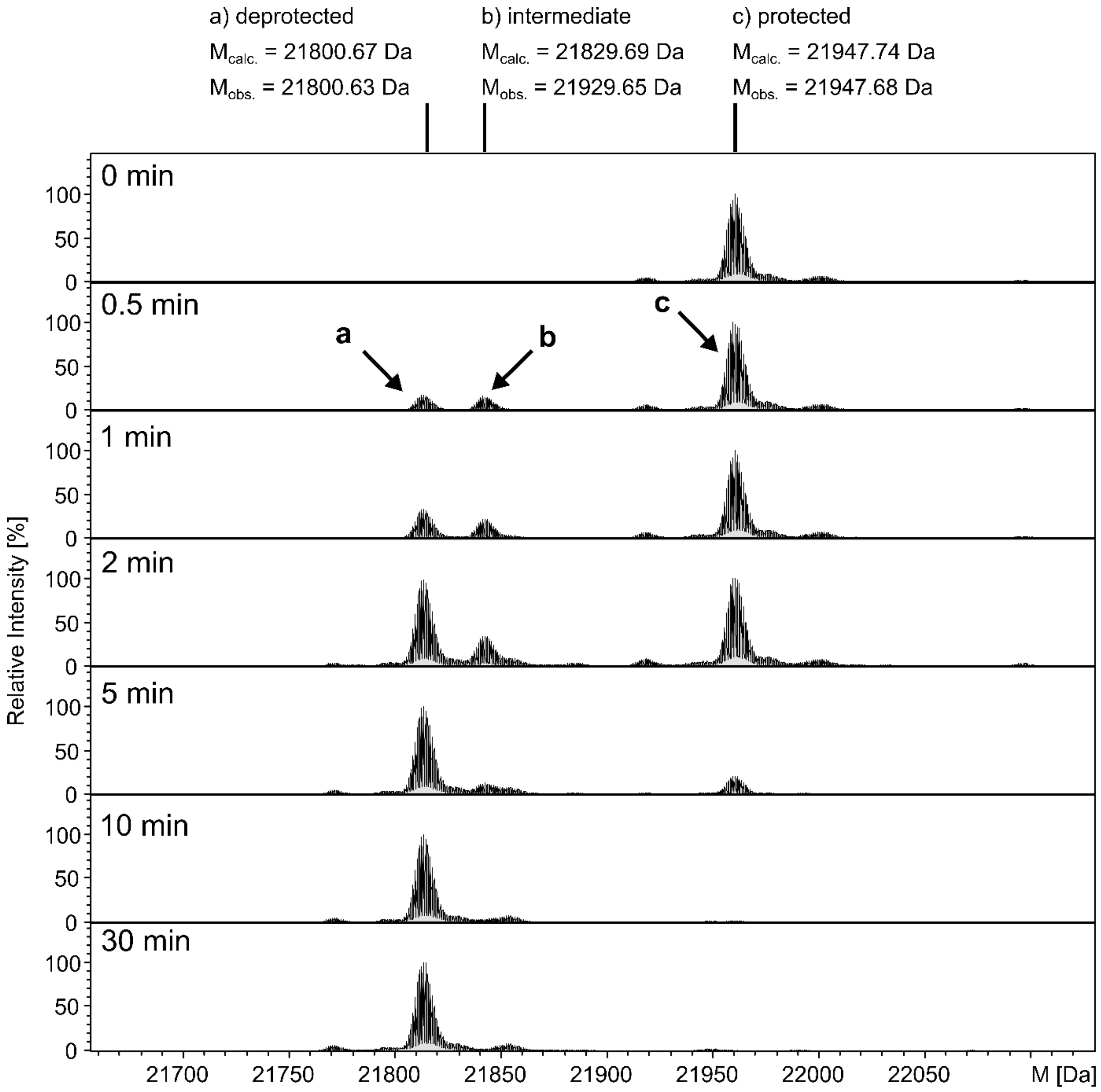

Figure S3: ESI-MS analysis of HcP deprotection. DiSUMO-I (**2**) (10 µM) was deprotected with PGA-His_6 (0.01 eq) and aliquots removed at the indicated time points were analyzed by ESI-MS. Formation and decomposition of the thioaminal intermediate (b) was observed within the first 5 min of reaction. $M_{obs.}$ is exemplarily shown for reaction products at time point 0.5 min.

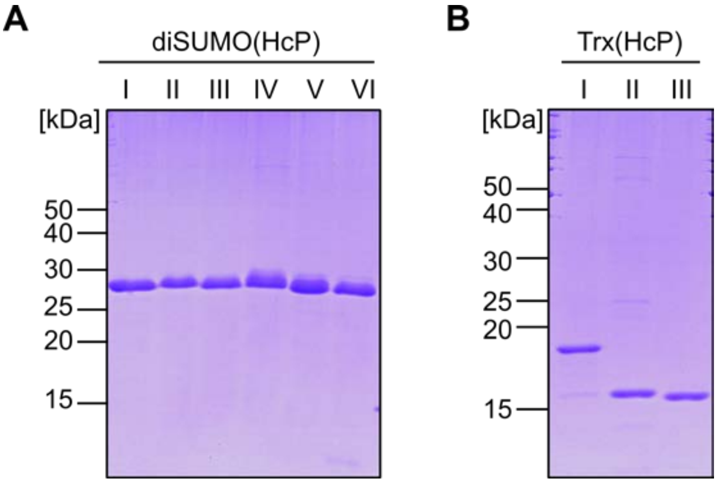

Figure S4: Characterization of purified proteins. Shown are Coomassie-stained SDS-PAGE gel analyses of A) the diSUMO(HcP) constructs and B) the Trx(HcP) constructs as presented in Figures 2B and C.

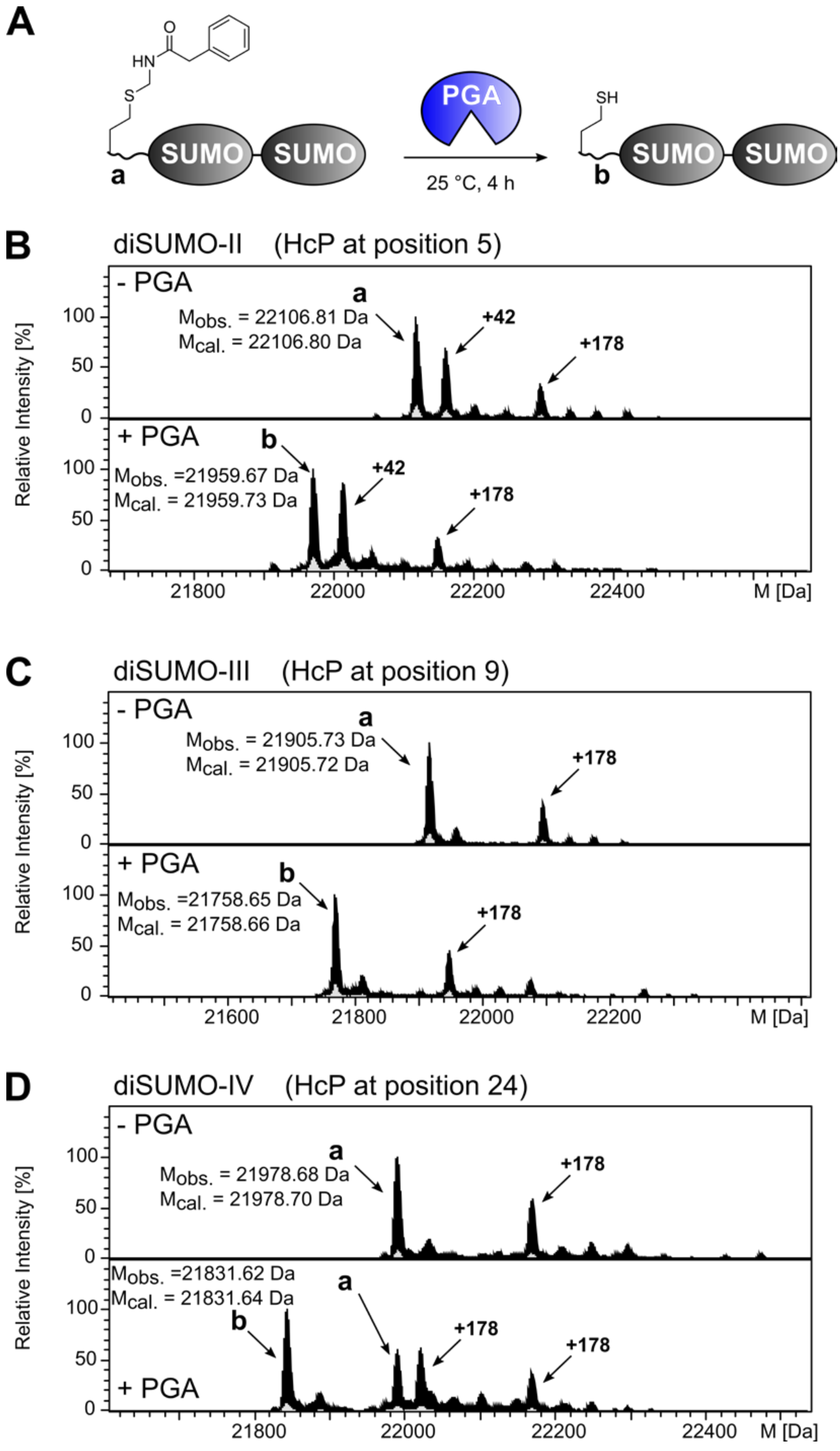

A
HN
O
S
SH
PGA
SUMO
SUMO
a
b
25 °C, 4 h
B
diSUMO-II (HcP at position 5)
- PGA
+ PGA
Relative Intensity [%]
$M_{obs.}$ = 22106.81 Da
$M_{cal.}$ = 22106.80 Da
+42
+178
$M_{obs.}$ =21959.67 Da
$M_{cal.}$ = 21959.73 Da
M [Da]
C
diSUMO-III (HcP at position 9)
$M_{obs.}$ = 21905.73 Da
$M_{cal.}$ = 21905.72 Da
$M_{obs.}$ =21758.65 Da
$M_{cal.}$ = 21758.66 Da
D
diSUMO-IV (HcP at position 24)
$M_{obs.}$ = 21978.68 Da
$M_{cal.}$ = 21978.70 Da
$M_{obs.}$ =21831.62 Da
$M_{cal.}$ = 21831.64 Da

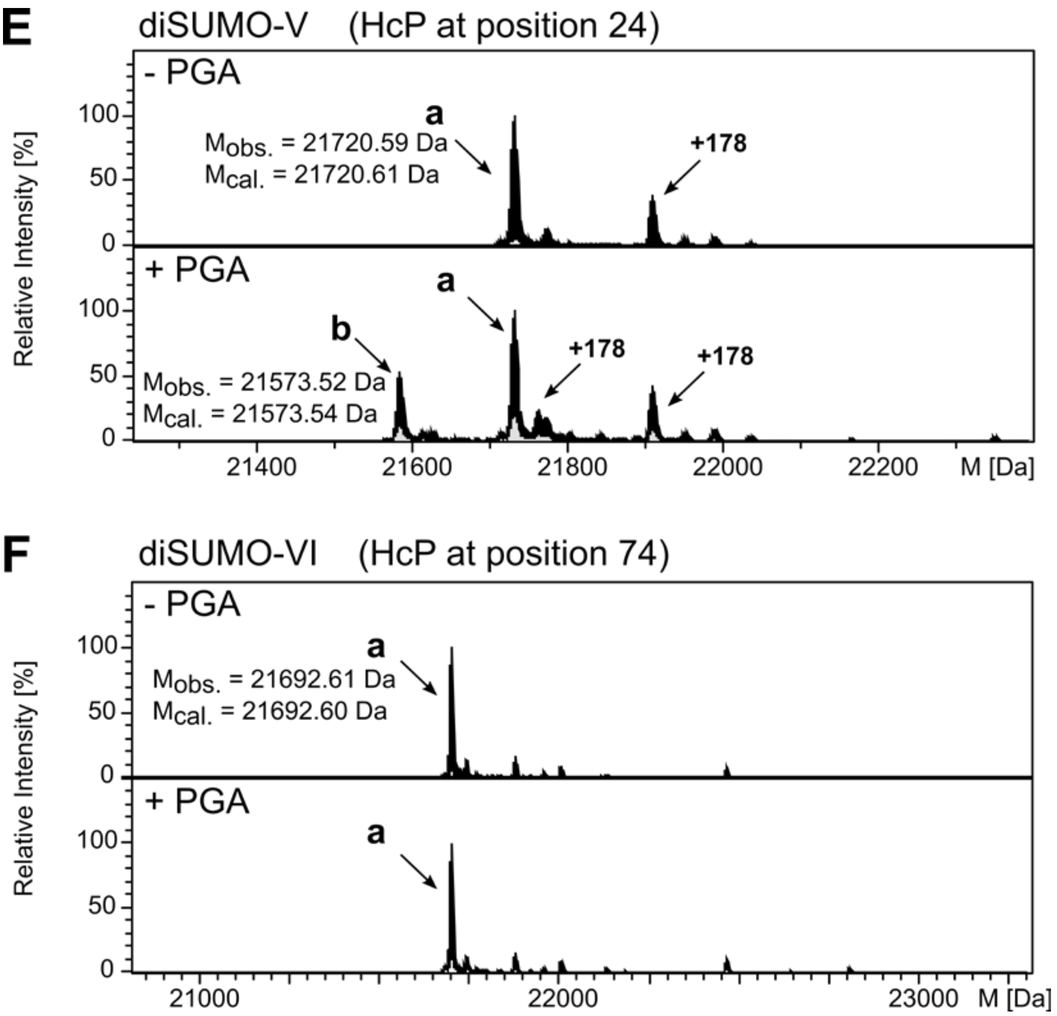

Figure S5: ESI-MS analysis of HcP deprotection. A) General scheme of the reaction. a = protected form; b = deprotected form. B-F) The indicated diSUMO(HcP) constructs (10 µM) were incubated with or without 0.1 eq PGA-His$_6$ for 4 h at 25 °C and then analyzed by ESI-MS. Modifications of +42 Da and +178 Da at varying levels were found depending on the proteins N-terminal sequence, which however, did not interfere with the PGA-mediated deprotection of HcP or subsequent bioconjugation. Proteins with an N-terminal His-tag can give rise to covalent modifications in *E. coli* with the +178 Da corresponding to the previously described α-*N*-gluconoylation.[1] HcP positions refer to numbering without the start methionine.

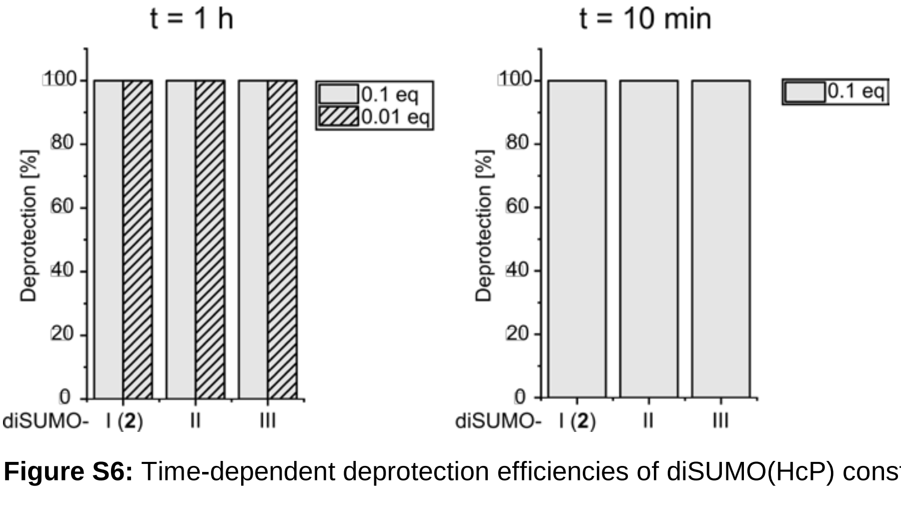

Figure S6: Time-dependent deprotection efficiencies of diSUMO(HcP) constructs with PGA-His$_6$.

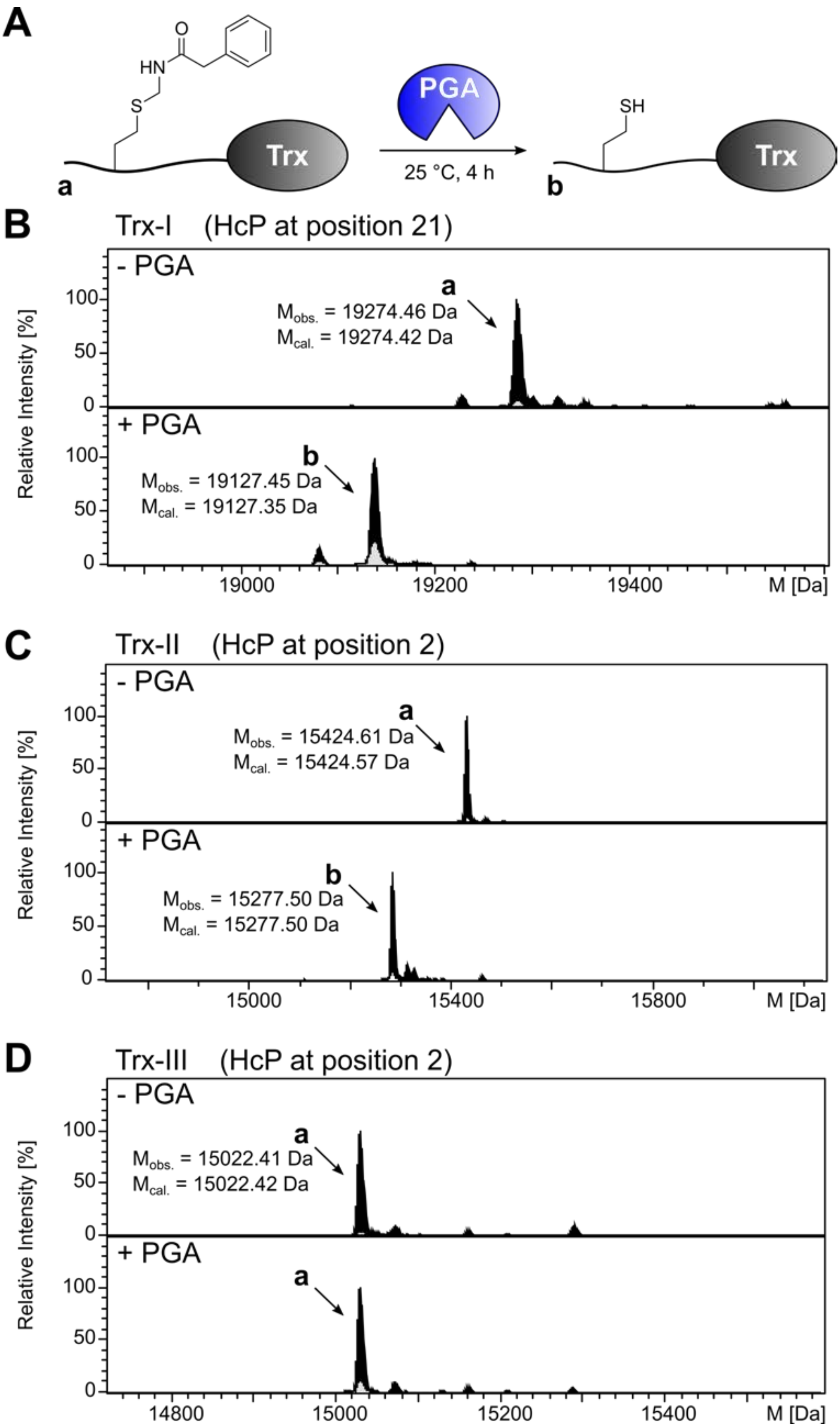

Figure S7: ESI-MS analysis of HcP deprotection. A) General scheme of the reaction. a = protected form; b = deprotected form. B-D) The indicated Trx(HcP) constructs (10 µM) were incubated with or without 0.1 eq PGA-His_6 for 4 h at 25 °C and then analyzed by ESI-MS. HcP positions refer to numbering without the start methionine.

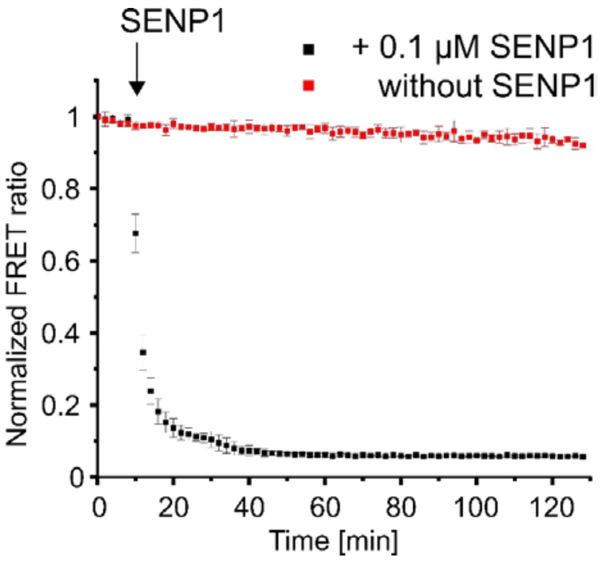

Figure S8: SENP1 cleavage assay of diSUMO FRET sensor. Protein **2*** (2 µM) was incubated with or without addition of SENP1 (0.1 µM) 10 min after start of measurement.

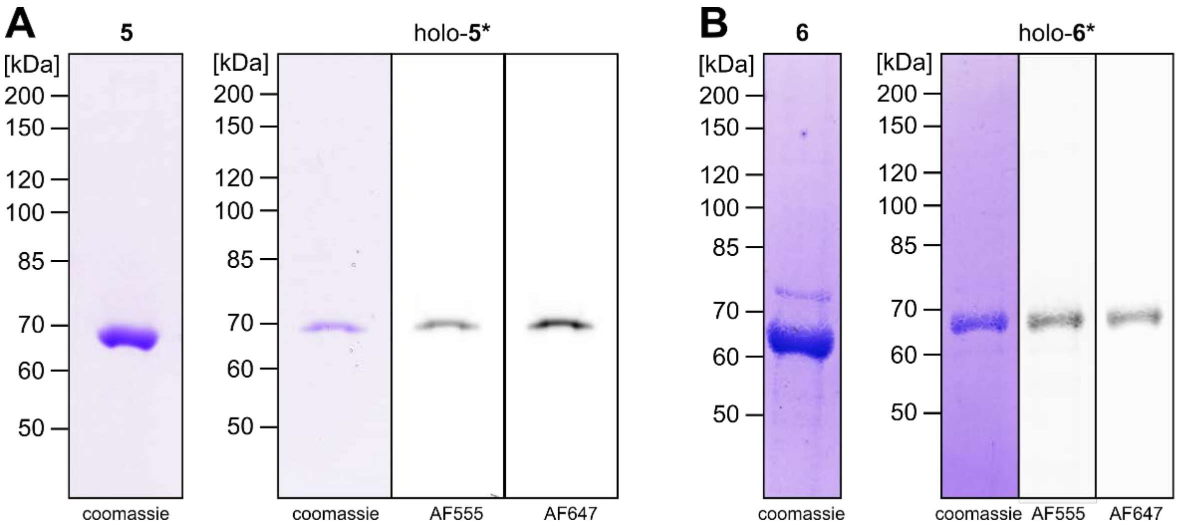

Figure S9: SDS-PAGE analysis of NRPS proteins. A) Purified Cys-HcP construct **5** (left panel) and dually-labeled FRET sensor holo-**5*** (right panels). B) Purified Cys-Cys construct **6** (left panel) and dually-labeled FRET sensor holo-**6*** (right panels). The fluorescence images to detect the AF555 and AF647 fluorophores were recorded with excitation at λ = 532 nm and λ = 635 nm, respectively.

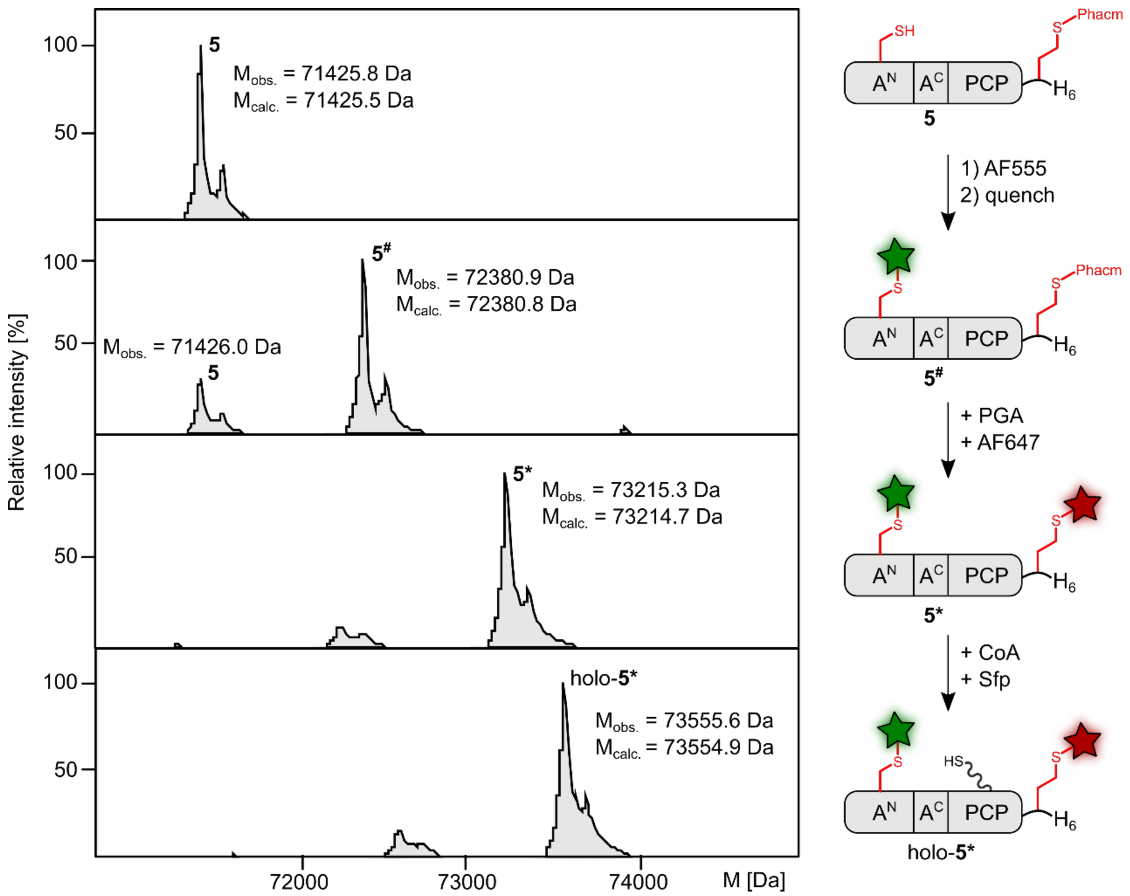

Figure S10: ESI-MS analysis of regioselective dual-labeling procedure of Cys-HcP construct **5**. As indicated in the scheme, the protein was first labeled with AF555 and quenched with DTT to give **5**$^{\#}$. Secondly, HcP was deprotected by addition of PGA-SBP and *in situ* labeled with AF647 to give **5***. Finally, the labeled protein was ppantylated into the holo-form to give the active FRET sensor holo-**5*** (ca. 91%). The left panel shows an ESI-MS analysis at each stage of the procedure.

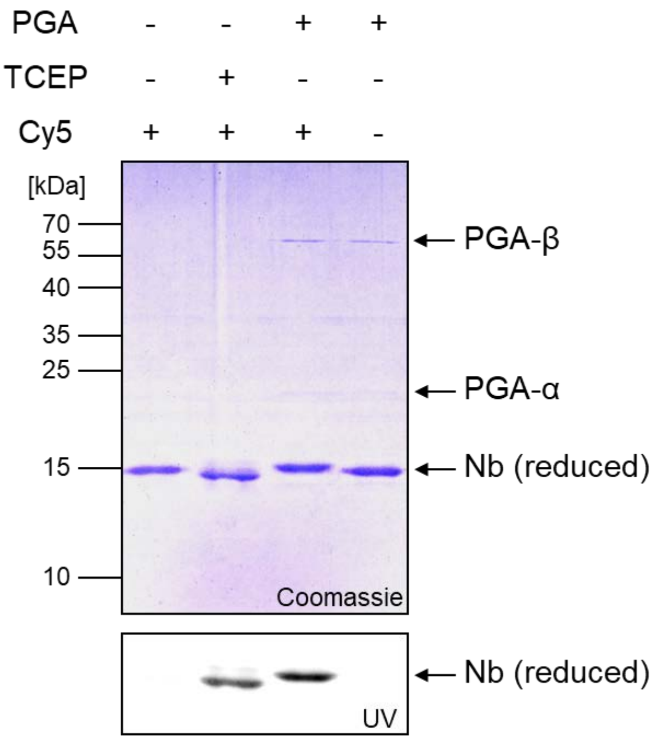

Figure S11: Further analysis of dimeric nanobody construct **7**-S-S-**7**. Shown are bioconjugation reactions analyzed using an SDS-PAGE gel. The protein (10 µM) was incubated with PGA-His$_6$ (0.01 eq), TCEP (10 eq) and Cy5 maleimide (5 eq), as indicated, for 1 h at 25 °C. UV illumination of the gel reports on the bioconjugation with Cy5 (bottom panel). Lane 1 (from left to right) shows that the disulfide was formed quantitatively, as no reaction with Cy5 maleimide occurred under these conditions. Following addition of TCEP (lane 2), the disulfide is reduced and Cy5 bioconjugated on the reduced Cys residue. Lanes 3 and 4 show that HcP deprotection, mediated by PGA-His$_6$, leads to a free thiol (Hcy) that undergoes bioconjugation with Cy5 maleimide. Together, these results provide independent evidence for the preservation of disulfide bonds during HcP deprotection and bioconjugation and further underlines the regioselectivity of the new dual labeling strategy. Note that the disulfide-linked homodimeric nanobody **7**-S-S-**7** is reduced to the monomers during SDS-PAGE sample preparation.

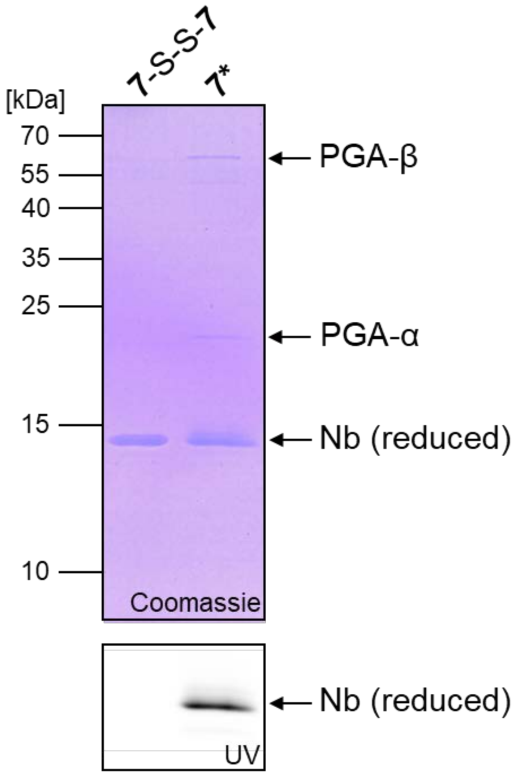

Figure S12: SDS-PAGE analysis of regioselective dual labeling of a nanobody. Shown is the same gel under UV illumination (bottom panel) and following Coomassie-staining (top panel). Left lane: purified **7**-S-S-**7**. Right lane: Deprotection with PGA-His_6 (0.02 eq) and *in situ* labeling with biotin maleimide (10 eq) for 1 h at 25 °C converted the protein into **7**$^{\#}$-S-S-**7**$^{\#}$. After quenching with DTT (1 mM) and subsequent removal of the DTT by dialysis, TCEP (10 eq) and Cy5 maleimide (5 eq, 1 h, 25 °C) were added to give **7***.

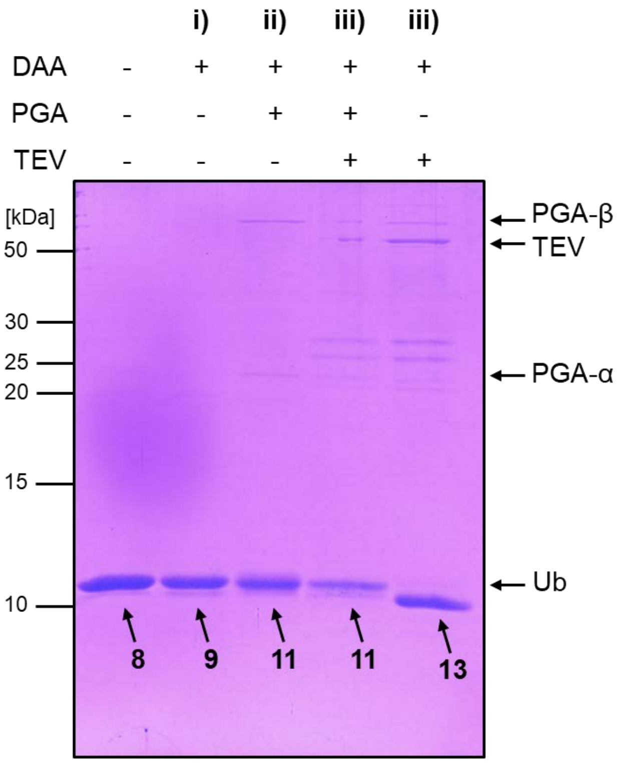

Figure S13: SDS-PAGE analysis of intramolecular crosslinking using a latent thiol group. The analyzed protein fractions correspond to the steps of the reaction scheme shown in Figure 6A of the main text. Importantly, intramolecularly crosslinked **11** is resistant to TEV cleavage, whereas linear **9** with the protected latent thiol group of HcP is quantitatively cleaved by TEV protease. PGA-His_6 was used for these experiments. DAA = dibromoadipic amide.

Chemical synthesis

Chemicals were purchased from Sigma-Aldrich, Thermo Fisher Scientific, Carl Roth, ChemShuttle, AppliChem, Acros Organics and Fluka. NMR-Spectra were measured on Bruker Avance II 300 and Bruker Avance II 400 spectrometers. Chemical shifts are referenced to the residual solvent signal. Peak multiplicity is abbreviated as follows: s = singulet, d = doublet, t = triplet, m = multiplet, td = triplet in doublet. Mass spectra were measured on MicroTof ESI from Bruker Daltonics.

N-(Hydroxymethyl)phenylacetamide

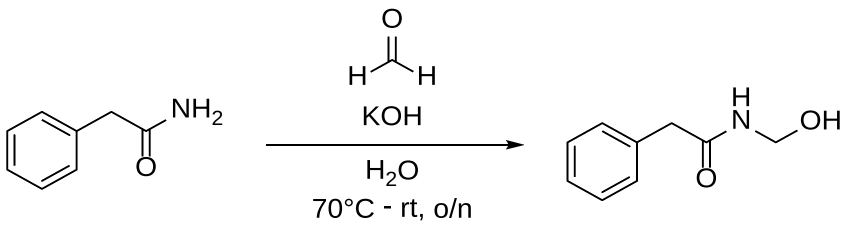

To a flask with phenylacetamide (4.0 g, 29.6 mmol, 1 eq) were added 30% formaldehyde solution (3 mL, 32.6 mmol, 1.1 eq), H_2O (3 mL) and KOH (185 mg, 3.30 mmol, 0.1 eq). The suspension was heated to 70 °C for 10 min, thereby forming a clear solution. The mixture was cooled to rt and stirred overnight. DCM was added and the aqueous phase extracted with DCM (3x 10 mL). The combined organic phases were dried over Na_2SO_4 and concentrated under reduced pressure to yield the product as white solid (4.1 g, 83%).

^{1}H-NMR (300 MHz, DMSO-d_6): δ = 8.63 (t, J = 6.5 Hz, 1H), 7.37 – 7.13 (m, 5H), 5.57 (t, J = 6.8 Hz, 1H), 4.50 (t, J = 6.6 Hz, 2H), 3.41 (s, 2H).

MS (ESI), *m/z* calcd for $C_9H_{11}NO_2+H^+$: 166.1 [M+H]$^+$; observed: 166.1.

The NMR data is in agreement with the literature.[2]

N-Boc-L-homocysteine methyl ester

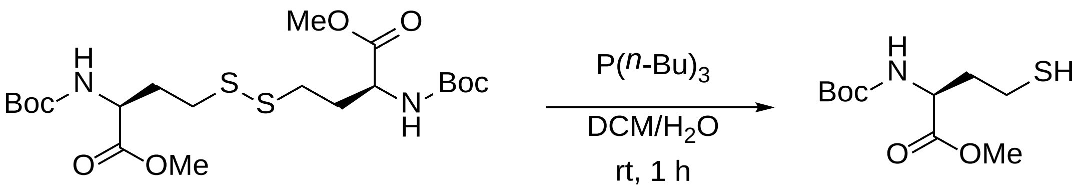

The reaction procedure was taken from Lherbet *et al.*.[3] *N*-Boc-L-homocystine methyl ester (2.5 g, 5.03 mmol, 1 eq) was dissolved in DCM (22 mL) under Ar. H_2O (1 mL) and tri-(*n*-butyl)phosphine (1.37 mL, 5.54 mmol, 1.1 eq) were added and the mixture stirred for 1 h at rt. Then, the organic phase was isolated, dried over Na_2SO_4 and concentrated under reduced pressure. The crude product was purified by column chromatography (5-25% EtOAc/cyclohexane) to yield an oil (1.8 g, 70%).

^{1}H-NMR (400 MHz, DMSO-d_6): δ = 7.28 (d, J = 7.9 Hz, 1H), 4.13 (td, J = 8.0, 6.3 Hz, 1H), 3.62 (s, 3H), 2.58 – 2.39 (m, 2H), 2.34 (t, J = 8.0 Hz, 1H), 1.91 – 1.81 (m, 2H), 1.38 (s, 9H).

HRMS (ESI), *m/z* calcd for $C_{10}H_{19}NO_4S+Na^+$: 272.0933 [M+Na]$^+$; observed: 272.0939.

The following reaction procedures were adapted vom Uprety *et al.*.[4]

Boc-L-Hcy(Phacm)-OMe

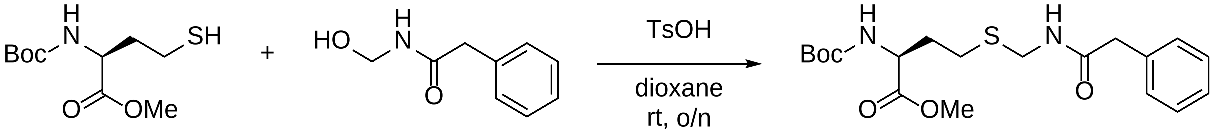

N-Boc-L-homocysteine methyl ester (1.8 g, 7.32 mmol, 1 eq) was dissolved in dry dioxane (22 mL) under Ar. *N*-(Hydroxymethyl)phenylacetamide (1.8 g, 11.0 mmol, 1.5 eq) and toluenesulfonic acid monohydrate (111 mg, 0.59 mmol, 0.08 eq) were added and the mixture was stirred overnight at rt. (Note: white solid formed overnight is side product.) EtOAc (100 mL) was added, the organic phase was washed with saturated aqueous $NaHCO_3$, dried over Na_2SO_4 and concentrated under reduced pressure. Purification by column chromatography (10-50% EtOAc/cyclohexane) yielded the product as an oil (2.2 g, 76%).

^{1}H-NMR (400 MHz, DMSOd$_6$): δ = 8.68 (t, J = 6.3 Hz, 1H), 7.34 – 7.16 (m, 5H), 4.28 – 4.13 (m, 2H), 4.09 – 4.03 (m, 1H), 3.62 (s, 3H), 3.43 (s, 2H), 2.63 – 2.50 (m, 2H), 1.95 – 1.74 (m, 2H), 1.38 (s, 9H).

HRMS (ESI), *m/z* calcd for $C_{19}H_{28}N_2O_5S$+Na^+: 419.1617 [M+Na]$^+$; observed: 419.1611

Boc-L-Hcy(Phacm)-OH

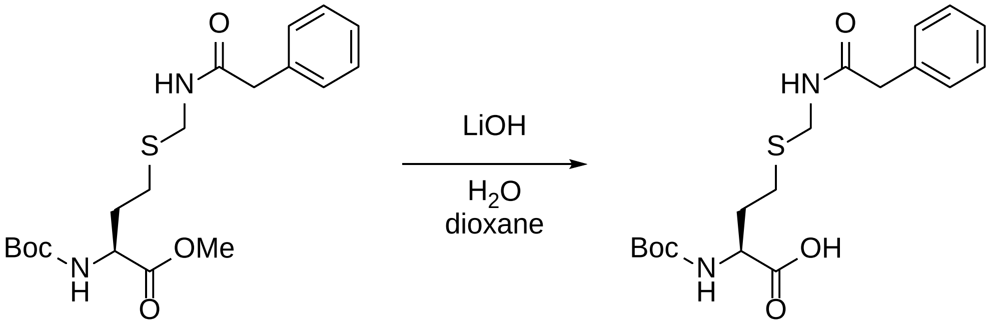

Boc-L-Hcy(Phacm)-OMe (2.2 g, 5.56 mmol, 1 eq) was dissolved in dioxane (12.5 mL) under Ar and cooled to 0 °C. 2 M $LiOH_{aq}$ (12.5 mL) was added and the solution was stirred for 1 h at 0 °C. The mixture was diluted with H_2O (50 mL) and washed with Et_2O (50 mL). The aqueous phase was acidified to pH = 2 - 3 with 1 M HCl and then extracted with EtOAc (3x 50 mL). The combined organic phases were washed with brine, dried over Na_2SO_4 and concentrated under reduced pressure to give the product as white solid (1.6 g, 73%).

HRMS (ESI), *m/z* calcd for $C_{18}H_{26}N_2O_5S$+Na^+: 405.1460 [M+Na]$^+$; observed: 405.1457

H-L-Hcy(Phacm)-OH (HcP, 1)

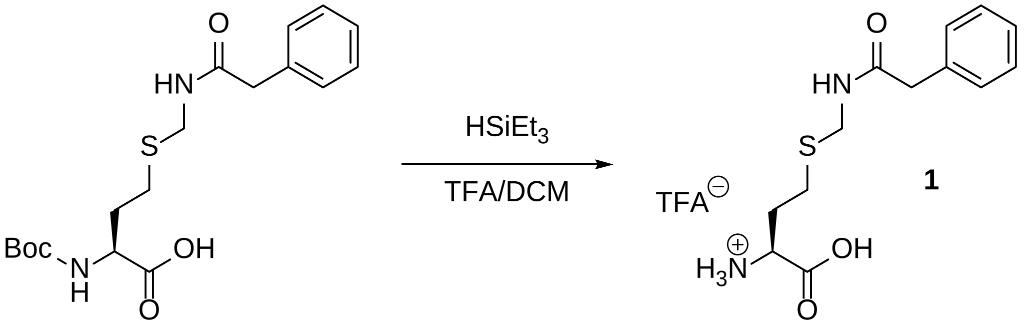

Boc-L-Hcy(Phacm)-OH (1.6 g, 4.09 mmol, 1 eq) was dissolved in DCM (10 mL) and TFA (10 mL) under Ar. Triethylsilane was added (0.78 mL, 4.90 mmol, 1.2 eq) and the reaction stirred at rt for 45 min, after which volatiles were removed under reduced pressure. The residue was redissolved in MeOH (10 mL) and concentrated again (3x). The crude product was dissolved in a small amount of MeOH and slowly added to cold Et_2O (80 mL). The precipitate was washed with Et_2O (2x 50 mL) to yield TFA salt **1** as white solid (926 mg, 80%).

^{1}H NMR (400 MHz, DMSO-d_6 + TFA): δ = 8.70 (t, *J* = 6.3 Hz, 1H), 8.23 (d, *J* = 5.7 Hz, 3H), 7.32 – 7.15 (m, 5H), 4.30 – 4.18 (m, 2H), 3.99 – 3.89 (m, 1H), 3.44 (s, 2H), 2.71 – 2.58 (m, 2H), 2.17 – 1.91 (m, 2H).

^{13}C NMR (101 MHz, DMSO-d_6 + TFA): δ = 171.18, 170.82, 136.34, 129.29, 128.52, 126.72, 51.26, 42.54, 30.90, 30.33, 25.78.

HRMS (ESI), *m/z* calcd for $C_{13}H_{18}N_2O_3S$+Na^+: 305.0936 [*M*+Na]$^+$; observed: 305.0930

Biochemical Methods

Expression and purification of proteins with HcP

For incorporation of HcP (**1**) by *amber* stop codon suppression, *E. coli* BL21 (DE3) gold cells were co-transformed with the respective plasmids coding for the target protein and mutant *Mb* PylRS(Y271M, L274A, C313A)/tRNA pair.[5, 6] Lysogeny broth (LB) medium was inoculated with overnight cultures and the appropriate antibiotics were added (ampicillin 100 µg/mL, kanamycin 50 µg/mL and chloramphenicol 34 µg/mL). Cells were grown at 37°C until OD_{600} = 0.6 – 0.8 was reached. LB cultures were then pre-incubated with HcP (2 mM) for 15 - 30 min prior to addition of L-(+)-arabinose (0.2% w/v)) and IPTG (0.4 mM) for induction (for thioredoxin constructs only arabinose was added). Cells were further incubated for 4 h at 37°C (diSUMO, Trx and Ub constructs) or 28°C (NRPS and Nb constructs). Next, cells were harvested by centrifugation (4000 rpm, 15 min, 4°C) and cell pellets were resuspended in Ni-NTA buffer (50 mM Tris, 300 mM NaCl, pH = 7.5 / 8). Cell lysis was performed using sonication (SONOPLUS, Bandelin) and remaining cell debris was removed by centrifugation. Protein purification from the soluble fraction was performed using Ni-NTA affinity chromatography and gravity flow columns. Ni-NTA resins (Cube Biotech) were equilibrated with Ni-NTA buffer containing 20 mM imidazole prior to addition of lysate. Resins were washed with Ni-NTA buffer with 20 mM imidazole and His-tagged target protein was eluted with Ni-NTA buffer containing 250 mM imidazole. Purified protein was dialyzed in Ni-NTA buffer in three steps. The last

dialysis step was performed with buffer additionally containing glycerol (10%). diSUMO-V was purified with a yield of 30.2 mg/L and for diSUMO-VI a yield of 26.0 mg/L was obtained.

Recombinant expression and purification of PGA

For production of PGA-His_6 and PGA-SBP (Streptavidin-Binding Peptide) *E. coli* BL21 (DE3) gold cells were transformed with the encoding plasmid.[7] LB medium containing 2 mM $CaCl_2$ and kanamycin (50 µg/mL) was inoculated with an overnight culture. Cells were grown until OD = 1 and induced with IPTG (1 mM). Cells were cultivated for another 4h at 28°C and harvested by centrifugation (4000 rpm, 15 min, 4°C). For purification of PGA-His_6 and PGA-SBP the cell pellets were resuspended in Ni-NTA buffer (pH = 7.5) or buffer W (100 mM Tris, 150 mM NaCl), pH = 8). Cell lysis was performed using an emulsifier (EmulsiFlex-C5, Avestin). Protein purification from the soluble fraction was performed by Ni-NTA affinity chromatography or SBP-tag affinity chromatography using 2.5 % glycerol in washing buffers and elution buffers.[7] Purified protein was dialyzed in Ni-NTA buffer in three steps (2 mM $CaCl_2$ each, 2.5 %, 2.5 % and 10% glycerol). Unless otherwise stated PGA-His_6 was used in the following experiments.

Western Blot

To detect His-tagged proteins anti-6x His epitope tag (rabbit, ROCKLAND) was used as primary antibody. A corresponding HRP-tagged Anti-rabbit (Dako) was utilized as second antibody. Detection by chemiluminescence was performed with ECL Western Blotting Analysis System (GE Healthcare).

HcP deprotection to reveal the latent thiol group

If not indicated otherwise, deprotection assays were performed by diluting HcP-containing proteins to a concentration of 10 µM in Ni-NTA buffer (pH = 7.5). PGA was added in varying concentrations and the solution incubated at 25 °C. The reaction was stopped by addition of formic acid to reach pH = 1 – 2. Samples were then analyzed as intact proteins by LC-MS. Time-course measurements of deprotection of **2** (Figure 1E) and deprotection assays of diSUMO and Trx constructs (Figures 2B and C) were performed in two technical repeats.

Mass spectrometry of intact proteins

For mass analyses of intact proteins an UltiMate™ 3000 RS system (Thermo Fisher Scientific Inc., MA, USA) was connected to a maXis II UHR-TOF mass spectrometer (Bruker Daltonik GmbH, Bremen, Germany). A standard ESI source (Apollo, Bruker Daltonik GmbH, Bremen, Germany). Samples were acidified by formic acid to reach a pH of 1 – 2. Except for Trx, Nb and Ub, samples were reduced for 10 min by TCEP (2 mM) prior to MS analysis to prevent inhomogeneity. After centrifugation (14000 rpm, 2 min, 4°C) samples were injected to LC-MS. A C4 column (Advance Bio RP-mAb C4, 2.1 mm x 50 mm, 3.5 µm, Agilent Technologies, Waldbronn, Germany) was used at a flow rate of 0.6 mL/min with eluents A and B (eluent A: 0.1% formic acid in H_2O; eluent B: 0.1% formic acid in acetonitrile). A desalting period (7 min, 5% B) was performed, followed by a steep gradient (5-60% B in 2 min). As settings for MS capillary voltage of 4500 V, end-plate offset of 500 V, a dry temperature of T=200°C and mass range of m/z 300-3000 were choosen. The nebulizer was set at 3.5 bar and flow rate of dry gas was 8.0 L/min. DataAnalysis 4.4 (Bruker Daltonik GmbH, Bremen, Germany) was used as analysis software. The software includes a MaxEnt algorithm, that was used for deconvolution.

Dual labeling strategy for diSUMO FRET sensor

HcP-containing diSUMO protein **2** (30 µM) was reduced by DTT (1 eq) in degased buffer Ni-NTA buffer (pH = 7.5) for 30 min and labeled with AlexaFluor 555 (AF555) maleimide (10 eq) for 45 min at 25°C. The reaction was stopped by addition of DTT (100 eq) and dialyzed in Ni-NTA buffer (pH = 7.5) to remove DTT. The HcP-containing diSUMO-AF555 **2**$^{\#}$ was then deprotected by PGA-SBP (0.01 eq) to reveal the Hcy thiol group and *in situ* bioconjugated with AlexaFluor 647 (AF647) maleimide (10 eq) for 45 min at 25°C, without adding a reducing agent. The reaction was quenched by addition of DTT (100 eq) and the modified protein was purified by Ni-NTA-affinity column chromatography, thereby removing excess fluorophore and PGA-SBP. The purified diSUMO FRET sensor **2*** was dialyzed in PBS buffer (pH = 7.4).

diSUMO cleavage with SENP1 analyzed by SDS-PAGE

For SDS-PAGE analysis diSUMO FRET sensor **2*** (10 µM) was incubated with and without the SUMO protease SENP1 (0.1 µM) for 2h at 25°C in PBS buffer (pH = 7.4). The reactions were stopped by addition of SDS-buffer and heating to 98°C for 10 min. Samples were visualized by UV illumination (excitation: 609 nm (AF647); 535 nm (AF555)) using Intas ECL Chemostar and by Coomassie Brilliant Blue staining. For LC-MS analysis of the cleavage reaction products, the reaction was stopped by heat (98°C) and acidified with formic acid to reach pH = 1 - 2.

diSUMO cleavage with SenP1 analyzed by FRET spectroscopy

FRET measurements of diSUMO FRET sensor **2*** (2 µM) were conducted in presence of 2 mM TCEP and 0.1% Tween in PBS buffer (pH = 7.4). Spectroscopic analysis of the SENP1 cleavage assay was performed with a Tecan plate-reader (Infinite® M1000 PRO) using 384-well microplates (Greiner bio-one, PS, Flat Bottom, non-binding, Black). Emission spectra (ex.: 520 nm) of **2*** incubated with or without SENP1 for 2h at 25°C were measured and corrected for direct excitation of the acceptor dye. For this purpose the emission spectrum of a diSUMO control protein labeled only with the acceptor dye AF647 (ex.: 520 nm) was substracted from the emission spectrum of **2***. For SENP1 cleavage assay of **2*** the donor dye AF555 was excited by 520 nm. SENP1 was added 10 min after beginning of the recording. Emission of the donor dye was measured at 570 nm (I_D) and emission of the acceptor dye at 665 nm (I_A). The FRET ratio (I_A / I_D) was corrected for direct excitation of the acceptor and cross talk of the donor as well as for the buffer background. For cross talk determination, diSUMO was labeled with only the donor dye or only the acceptor dye. For direct excitation of the acceptor the only-acceptor labeled diSUMO was excited by 520 nm and its emission at 665 nm was measured. The donor cross talk was determined by excitation of the only-donor labeled diSUMO at 665 nm when excited by 520 nm. FRET ratio was normalized to the ratio at t = 0. Experiments were performed in three technical repeats (Figure S8).

Dual labeling to generate NRPS FRET sensors

The NRPS construct A-PCP(N152C/HcP-tag) **5** (20 µM) was reduced by TCEP (20 eq.) in buffer (pH = 7; 50 mM Tris, 300 mM NaCl) for 30 min (18 °C) and modified with Alexa Fluor 555 maleimide (10 eq.). The reaction was quenched with DTT (5 eq.) and dialyzed in Ni-NTA buffer (pH = 7.5; 50 mM Tris, 300 mM NaCl) to remove excess DTT. Then PGA-SBP (0.5 eq.)

and Alexa Fluor 647 maleimide (10 eq.) were added and the reaction mixture was incubated for 2 h (25 °C) to effect an *in situ* bioconjugation of the latent thiol group of the revealed Hcy. To remove the excess fluorophore reagent and PGA-SBP the modified protein was purified by Ni-NTA-affinity column chromatography. The dually labeled protein was then converted into its active holo-form by addition of 4`-phosphopantetheinyl transferase Sfp (0.05 eq.), coenzyme A (100 eq.), $MgCl_2$ (10 mM) and TCEP (2 mM) and incubated for 2 h (RT) to give holo-**5***. The protein was dialyzed against assay buffer (50 mM HEPES, 100 mM NaCl, 1 mM EDTA, 10 mM $MgCl_2$, 10% glycerol, pH = 7) for subsequent FRET measurements. The NRPS construct A-PCP(N152C/Cys-tag) **6** was treated in a similar way except that Alexa Fluor 555 maleimide (5 eq.) and Alexa Fluor 647 maleimide (10 eq.) were added as a mixture to give the stochastic labeled active Cys-Cys construct holo-**6***.

FRET measurements with NRPS FRET sensors

FRET measurements with the NRPS constructs holo-**5*** and holo-**6*** were performed with a Tecan (Infinite® M1000 PRO) using 384-well microplates (Greiner bio-one, PS, Flat Bottom, non-binding, Black). holo-**5*** and holo-**6*** (0.3 µM) were mixed with and without ATP and L-Phe (2 mM each) in a total volume of 50 µL in assay buffer (50 mM HEPES, 100 mM NaCl, 1 mM EDTA, 10 mM $MgCl_2$, pH = 7) and incubated for 30 min before starting the measurement (25 °C). AF555 was excited at 520 nm and emission was measured from 560 to 800 nm (bandwidth = 5 nm). AF647 was excited at 650 nm and emission was measured from 660 to 800 nm. The obtained emissions at 570 nm (I_d) and at 674 nm (I_a) at donor excitation were normalized to the acceptor only emission at acceptor excitation, thus correcting for concentration fluctuations. The FRET ratio I_a/I_d was calculated and the values of six replicates averaged.

Dual labeling of a nanobody

Purified **7**-S-S-**7** (20 µM) in Ni-NTA buffer (pH = 7.5) was incubated with PGA-His_6 (0.02 eq) and biotin maleimide (5 eq) for 1 h at 25 °C to give **7$^{\#}$**-S-S-**7$^{\#}$**. Excess maleimide reagent was quenched with DTT (1 mM) and removed by dialysis into fresh Ni-NTA buffer (pH = 7.5) using VivaSpin 500 columns (5,000 MWCO; Sartorius) to yield **7$^{\#}$**. For subsequent cysteine labeling, TCEP (10 eq) and Cy5 maleimide (10 eq) were added. After 1 h at 25 °C, again DTT (1 mM) was added for quenching. Resulting dually labeled anti-EGFP nanobody **7*** was used for mammalian cell binding experiments without further purification.

Mammalian cell culture and nanobody binding assay

HeLa cells were cultured in EMEM supplemented with 10% fetal calf serum, 1% non-essential amino acids and 1% L-glutamine) at 37 °C and 5% CO_2. Half confluent cells were seeded in 35 mm dishes and used for transient transfection via calcium phosphate coprecipitation with plasmid pMBH63, encoding HA-EGFP-Trx-TMD-mCherry with a signal sequence for transport to the plasma membrane.[8] After 24 h of incubation, the binding assay was performed. Cells were washed three times with PBS and dually labeled Nb **7*** was added to 1 mL of the fresh medium in a final concentration of 10 nM. After 5 min incubation at 25 °C, cells were washed three times with PBS and iFluor405-streptavidin conjugate was added to 1 mL of fresh medium in a final concentration of 30 nM. Again, after 5 min incubation at 25 °C, cells were washed three times with PBS and fixed by addition of 500 µL paraformaldehyde (4%).

Confocal laser scanning microscopy

Fixed cells were mounted on coverslips with Aqua/Poly-Mount solution (Polysciences). Confocal microscopy was performed using a 63X water-immersion objective lens on a Leica DMi8 system.

Enzyme-activated protein crosslinking using the latent thiol group

Purified **8** was reduced with DTT and the reductant removed by dialysis. **8** (20 µM) was then incubated with dibromoadipic amide (DAA, 10 eq) in Ni-NTA buffer (pH = 9.2) at 37 °C overnight. Resulting protein **9** was purified by dialysis into Ni-NTA buffer (pH = 8.8) using VivaSpin 500 columns (5,000 MWCO; Sartorius). PGA-His$_6$ (0.01 eq) and TCEP (5 eq) were added for overnight (25 °C) deprotection and cyclization. The prolonged reaction time and addition of TCEP were found to be required because of the slow rate of the addition reaction. Finally, **9** and the potential mixture of **10** and **11** were incubated with TEV protease (0.1 eq) for 3 h at 25 °C.

Supporting Table

Table S1. Recombinant proteins and expression vectors used in this study

protein construct	plasmid	vector, marker	amino acid sequence	ref.
**diSUMO-I (2)**<br>MG**HcP**G-His$_6$-TEV-SUMO2(C48A)-(Δ10)SUMO2(C48A, R61C)	pMR50	pET28a<br>Kan	MG**HcP**GHHHHHHENLYFQGADEKPKEGVKTENNDHINLKVAGQDGSVVQFKIKRHTPLSKLMKAYAERQGLSMRQIRFRFDGQPINETDTPAQLEMEDEDTIDVFQQQTGGKTENNDHINLKVAGQDGSVVQFKIKRHTPLSKLMKAYAERQGLSMRQIRFCFDGQPINETDTPAQLEMEDEDTIDVFQQQTGG	This work
**diSUMO-II**<br>MGSGG**HcP**G-His$_6$-TEV-SUMO2(C48A)-(Δ10)SUMO2(C48A, R61C)	pAA35	pET28a<br>Kan	MGSGG**HcP**GHHHHHHENLYFQGADEKPKEGVKTNNDHINLKVAGQDGSVVQFKIKRHTPLSKLMKAYAERQGLSMRQIRFRFDGQPINETDTPAQLEMEDEDTIDVFQQQTGGKTENNDHINLKVAGQDGSVVQFKIKRHTPLSKLMKAYAERQGLSMRQIRFCFDGQPINETDTPAQLEMEDEDTIDVFQQQTGG	This work
**diSUMO-III**<br>MG-His$_6$-G**HcP**-TEV-SUMO2(C48A)-(Δ10)SUMO2(C48A, R61C)	pAA36	pET28a<br>Kan	MGHHHHHHG**HcP**ENLYFQGADEKPKEGVKTENNDHINLKVAGQDGSVVQFKIKRHTPLSKLMKAYAERQGLSMRQIRFRFDGQPINETDTPAQLEMEDEDTIDVFQQQTGGKTENNDHINLKVAGQDGSVVQFKIKRHTPLSKLMKAYAERQGLSMRQIRFCFDGQPINETDTPAQLEMEDEDTIDVFQQQTGG	This work
**diSUMO-IV**<br>MG-His$_6$-TEV-SUMO2(K11**HcP**, +GGSG, C48A)-(Δ10)SUMO2(C48A, R61C)	pPM38	pET28a<br>Kan	MGHHHHHHENLYFQGADEKPKEGV**HcP**TENGGSGNDHINLKVAGQDGSVVQFKIKRHTPLSKLMKAYAERQGLSMRQIRFRFDGQPINETDTPAQLEMEDEDTIDVFQQQTGGKTENNDHINLKVAGQDGSVVQFKIKRHTPLSKLMKAYAERQGLSMRQIRFCFDGQPINETDTPAQLEMEDEDTIDVFQQQTGG	This work
**diSUMO-V**<br>MG-His$_6$-TEV-SUMO2(K11**HcP**, C48A)-(Δ10)SUMO2(C48A, R61C)	pAA20	pET28a<br>Kan	MGHHHHHHENLYFQGADEKPKEGV**HcP**TENNDHINLKVAGQDGSVVQFKIKRHTPLSKLMKAYAERQGLSMRQIRFRFDGQPINETDTPAQLEMEDEDTIDVFQQQTGGKTENNDHINLKVAGQDGSVVQFKIKRHTPLSKLMKAYAERQGLSMRQIRFCFDGQPINETDTPAQLEMEDEDTIDVFQQQTGG	This work
**diSUMO-VI**<br>MG-His$_6$-TEV-SUMO2(C48A, R61**HcP**)-(Δ10)SUMO2(C48A, R61C)	pLK44	pET28a<br>Kan	MGHHHHHHENLYFQGADEKPKEGVKTENNDHINLKVAGQDGSVVQFKIKRHTPLSKLMKAYAERQGLSMRQIRF**HcP**FDGQPINETDTPAQLEMEDEDTIDVFQQQTGGKTENNDHINLKVAGQDGSVVQFKIKRHTPLSKLMKAYAERQGLSMRQIRFCFDGQPINETDTPAQLEMEDEDTIDVFQQQTGG	[9]

**Trx-I**<br>Linker(**HcP**)-TrxA-Linker-His$_6$	pPM48	pBAD<br>Amp	MGIEGRISEFYIDTDSVVGDT**HcP**IDVSGKKMTIAEFYDS<br>TPDGDKIIHLTDDSFDTDVLKADGAILVDFWAEWCGPCK<br>MIAPILDEIADEYQGKLTVAKLNIDQNPGTAPKYGIRGIPT<br>LLLFKNGEVAATKVGALSKGQLKEFLDANLAGSGSGER<br>QHMDSPDLGTDDDDKHHHHHH	This work
**Trx-II**<br>MG**HcP**GGSGG-TrxA-Linker-His$_6$	pPM37	pBAD<br>Amp	MG**HcP**GGSGGSDKIIHLTDDSFDTDVLKADGAILVDFWA<br>EWCGPCKMIAPILDEIADEYQGKLTVAKLNIDQNPGTAP<br>KYGIRGIPTLLLFKNGEVAATKVGALSKGQLKEFLDANLA<br>GSGSGERQHMDSPDLGTDDDDKHHHHHH	This work
**Trx-III**<br>TrxA(S3**HcP**)-Linker-His$_6$	pPM33	pBAD<br>Amp	MG**HcP**DKIIHLTDDSFDTDVLKADGAILVDFWAEWCGPC<br>KMIAPILDEIADEYQGKLTVAKLNIDQNPGTAPKYGIRGIP<br>TLLLFKNGEVAATKVGALSKGQLKEFLDANLAGSGSGE<br>RQHMDSPDLGTDDDDKHHHHHH	This work
**GFP-enhancer nanobody (Nb)**<br>PelB-G**HcP**-GS-enhancerNB-Cys-His$_6$	pPM45	pET22b(+)<br>Amp	MKYLLPTAAAGLLLLAAQPAMAMDGG**HcP**GSGGSGKL<br>MAQVQLVESGGALVQPGGSLRLSCAASGFPVNRYSMR<br>WYRQAPGKEREWVAGMSSAGDRSSYEDSVKGRFTISR<br>DDARNTVYLQMNSLKPEDTAVYYCNVNVGFEYWGQGT<br>QVTVSSPDGCHHHHHH	This work and [10]
**GrsA-A-PCP(HcP)**<br><br>GrsA-A(C60F, C331A, C376S, N152C)-PCP-RSEAGV**HcP**TE-His$_6$	pMR36	pET28a<br>Kan	MVNSSKSILIHAQNKNGTHEEEQYLFAVNNTKAEYPRDK<br>TIHQLFEEQVSKRPNNVAIVFENEQLTYHELNVKANQLA<br>RIFIEKGIGKDTLVGIMMEKSIDLFIGILAVLKAGGAYVPIDI<br>EYPKERIQYILDDSQARMLLTQKHLVHLIHCIQFNGQVEI<br>FEEDTIKIREGTNLHVPSKSTDLAYVIYTSGTTGNPKGTM<br>LEHKGISNLKVFFENSLNVTEKDRIGQFASISFDASVWE<br>MFMALLTGASLYIILKDTINDFVKFEQYINQKEITVITLPPT<br>YVVHLDPERILSIQTLITAGSATSPSLVNKWKEKVTYINAY<br>GPTETTIAATTWVATKETIGHSVPIGAPIQNTQIYIVDENL<br>QLKSVGEAGELSIGGEGLARGYWKRPELTSQKFVDNPF<br>VPGEKLYKTGDQARWLSDGNIEYLGRIDNQVKIRGHRV<br>ELEEVESILLKHMYISETAVSVHKDHQEQPYLAAYFVSE<br>KHIPLEQLRQFSSEELPTYMIPSYFIQLDKMPLTSNGKID<br>RKQLPEPDLTFGMRVDYEAPRNEIEETLVTIWQDVLGIE<br>KIGIKDNFYALGGDSIKAIQVAARLHSYQLKLETKDLLKYP<br>TIDQLVHYIKDSKRRSEAGV**HcP**TEHHHHHH	This work and [11]
**GrsA-A-PCP**(**Cys**)<br><br>GrsA-A(C60F, C331A, C376S, N152C)-PCP-RSEAGV**C**TE-His$_6$	pAF14	pET28a<br>Kan	MVNSSKSILIHAQNKNGTHEEEQYLFAVNNTKAEYPRDK<br>TIHQLFEEQVSKRPNNVAIVFENEQLTYHELNVKANQLA<br>RIFIEKGIGKDTLVGIMMEKSIDLFIGILAVLKAGGAYVPIDI<br>EYPKERIQYILDDSQARMLLTQKHLVHLIHCIQFNGQVEI<br>FEEDTIKIREGTNLHVPSKSTDLAYVIYTSGTTGNPKGTM<br>LEHKGISNLKVFFENSLNVTEKDRIGQFASISFDASVWE<br>MFMALLTGASLYIILKDTINDFVKFEQYINQKEITVITLPPT<br>YVVHLDPERILSIQTLITAGSATSPSLVNKWKEKVTYINAY<br>GPTETTIAATTWVATKETIGHSVPIGAPIQNTQIYIVDENL<br>QLKSVGEAGELSIGGEGLARGYWKRPELTSQKFVDNPF<br>VPGEKLYKTGDQARWLSDGNIEYLGRIDNQVKIRGHRV<br>ELEEVESILLKHMYISETAVSVHKDHQEQPYLAAYFVSE<br>KHIPLEQLRQFSSEELPTYMIPSYFIQLDKMPLTSNGKID<br>RKQLPEPDLTFGMRVDYEAPRNEIEETLVTIWQDVLGIE<br>KIGIKDNFYALGGDSIKAIQVAARLHSYQLKLETKDLLKYP<br>TIDQLVHYIKDSKRRSEAGV**C**TEHHHHHH	This work and [11]
**PGA-His$_6$**<br><br>Heterodimeric protein (α and β subunits)-His$_6$	pETEH	pET24a(+)<br>Kan	MKNRNRMIVNCVTASLMYYWSLPALAEQSSSEIKIVRDE<br>YGMPHIYANDTWHLFYGYGYVVAQDRLFQMEMARRST<br>QGTVAEVLGKDFVKFDKDIRRNYWPDAIRAQIAALSPED<br>MSILQGYADGMNAWIDKVNTNPETLLPKQFNTFGFTPK<br>RWEPFDVAMIFVGTMANRFSDSTSEIDNLALLTALKDKY<br>GVSQGMAVFNQLKWLVNPSAPTTIAVQESNYPLKFNQQ<br>NSQTAALLPRYDLPAPMLDRPAKGADGALLALTAGKNR<br>ETIAAQFAQGGANGLAGYPTTSNMWVIGKSKAQDAKAI<br>MVNGPQFGWYAPAYTYGIGLHGAGYDVTGNTPFAYPG<br>LVFGHNGVISWGSTAGFGDDVDIFAERLSAEKPGYYLH<br>NGKWVKMLSREETITVKNGQAETFTVWRTVHGNILQTD<br>QTTQTAYAKSRAWDGKEVASLLAWTHQMKAKNWQEW<br>TQQAAKQALTINWYYADVNGNIGYVHTGAYPDRQSGH<br>DPRLPVPGTGKWDWKGLLPFEMNPKVYNPQSGYIANW<br>NNSPQKDYPASDLFAFLWGGADRVTEIDRLLEQKPRLT<br>ADQAWDVIRQTSRQDLNLRLFLPTLQAATSGLTQSDPR<br>RQLVETLTRWDGINLLNDDGKTWQQPGSAILNVWLTSM<br>LKRTVVAAVPMPFDKWYSASGYETTQDGPTGSLNISVG<br>AKILYEAVQGDKSPIPQAVDLFAGKPQQEVVLAALEDTW<br>ETLSKRYGNNVSNWKTPAMALTFRANNFFGVPQAAAE<br>ETRHQAEYQNRGTENDMIVFSPTTSDRPVLAWDVVAP<br>GQSGFIAPDGTVDKHYEDQLKMYENFGRKSLWLTKQD<br>VEAHKESQEVLHVQRHHHHHH	[7]
**PGA-SBP**	pPM36	pET24a(+)	MKNRNRMIVNCVTASLMYYWSLPALAEQSSSEIKIVRDE<br>YGMPHIYANDTWHLFYGYGYVVAQDRLFQMEMARRST	This work

Heterodimeric protein (α and β subunits)-SBP		Kan	QGTVAEVLGKDFVKFDKDIRRNYWPDAIRAQIAALSPED<br>MSILQGYADGMNAWIDKVNTNPETLLPKQFNTFGFTPK<br>RWEPFDVAMIFVGTMANRFSDSTSEIDNLALLTALKDKY<br>GVSQGMAVFNQLKWLVNPSAPTTIAVQESNYPLKFNQQ<br>NSQTAALLPRYDLPAPMLDRPAKGADGALLALTAGKNR<br>ETIAAQFAQGGANGLAGYPTTSNMWVIGKSKAQDAKAI<br>MVNGPQFGWYAPAYTYGIGLHGAGYDVTGNTPFAYPG<br>LVFGHNGVISWGSTAGFGDDVDIFAERLSAEKPGYYLH<br>NGKWVKMLSREETITVKNGQAETFTVWRTVHGNILQTD<br>QTTQTAYAKSRAWDGKEVASLLAWTHQMKAKNWQEW<br>TQQAAKQALTINWYYADVNGNIGYVHTGAYPDRQSGH<br>DPRLPVPGTGKWDWKGLLPFEMNPKVYNPQSGYIANW<br>NNSPQKDYPASDLFAFLWGGADRVTEIDRLLEQKPRLT<br>ADQAWDVIRQTSRQDLNLRLFLPTLQAATSGLTQSDPR<br>RQLVETLTRWDGINLLNDDGKTWQQPGSAILNVWLTSM<br>LKRTVVAAVPMPFDKWYSASGYETTQDGPTGSLNISVG<br>AKILYEAVQGDKSPIPQAVDLFAGKPQQEVVLAALEDTW<br>ETLSKRYGNNVSNWKTPAMALTFRANNFFGVPQAAAE<br>ETRHQAEYQNRGTENDMIVFSPTTSDRPVLAWDVVAP<br>GQSGFIAPDGTVDKHYEDQLKMYENFGRKSLWLTKQD<br>VEAHKESQEVLHVQRMDEKTTGWRGGHVVEGLAGELE<br>QLRARLEHHPQGQREP	and [7]
**SENP1**<br><br>GST-SENP1(E417-L644)	pGST-Senp1	pGEX4T-1<br>Amp		[12]

Supporting References

[1] K. F. Geoghegan, H. B. Dixon, P. J. Rosner, L. R. Hoth, A. J. Lanzetti, K. A. Borzilleri, E. S. Marr, L. H. Pezzullo, L. B. Martin, P. K. LeMotte, A. S. McColl, A. V. Kamath, and J. G. Stroh, *Anal Biochem* **1999**, *267*, 169.

[2] M. Royo, J. Alsina, E. Giralt, U. Slomcyznska, and F. Albericio, *J. Chem. Soc., Perkin Trans. 1* **1995**, 1095.

[3] C. Lherbet, and J. W. Keillor, *Org Biomol Chem* **2004**, *2*, 238.

[4] R. Uprety, J. Luo, J. Liu, Y. Naro, S. Samanta, and A. Deiters, *Chembiochem* **2014**, *15*, 1793.

[5] M. Cigler, T. G. Muller, D. Horn-Ghetko, M. K. von Wrisberg, M. Fottner, R. S. Goody, A. Itzen, M. P. Muller, and K. Lang, *Angew Chem Int Ed Engl* **2017**, *56*, 15737.

[6] M. Reille-Seroussi, S. V. Mayer, W. Dorner, K. Lang, and H. D. Mootz, *Chem Commun (Camb)* **2019**, *55*, 4793.

[7] T. Cheng, M. Chen, H. Zheng, J. Wang, S. Yang, and W. Jiang, *Protein Expr Purif* **2006**, *46*, 107.

[8] B. Jedlitzke, Z. Yilmaz, W. Dorner, and H. D. Mootz, *Angew Chem Int Ed Engl* **2020**, *59*, 1506.

[9] L. J. Kost, and H. D. Mootz, *Chembiochem* **2018**, *19*, 177.

[10] A. Kirchhofer, J. Helma, K. Schmidthals, C. Frauer, S. Cui, A. Karcher, M. Pellis, S. Muyldermans, C. S. Casas-Delucchi, M. C. Cardoso, H. Leonhardt, K. P. Hopfner, and U. Rothbauer, *Nat Struct Mol Biol* **2010**, *17*, 133.

[11] J. Alfermann, X. Sun, F. Mayerthaler, T. E. Morrell, E. Dehling, G. Volkmann, T. Komatsuzaki, H. Yang, and H. D. Mootz, *Nat Chem Biol* **2017**, *13*, 1009.

[12] A. Werner, M. C. Moutty, U. Moller, and F. Melchior, *Methods Mol Biol* **2009**, *497*, 187.

Appendix

^{1}H NMR spectrum of HcP (**1**) TFA salt

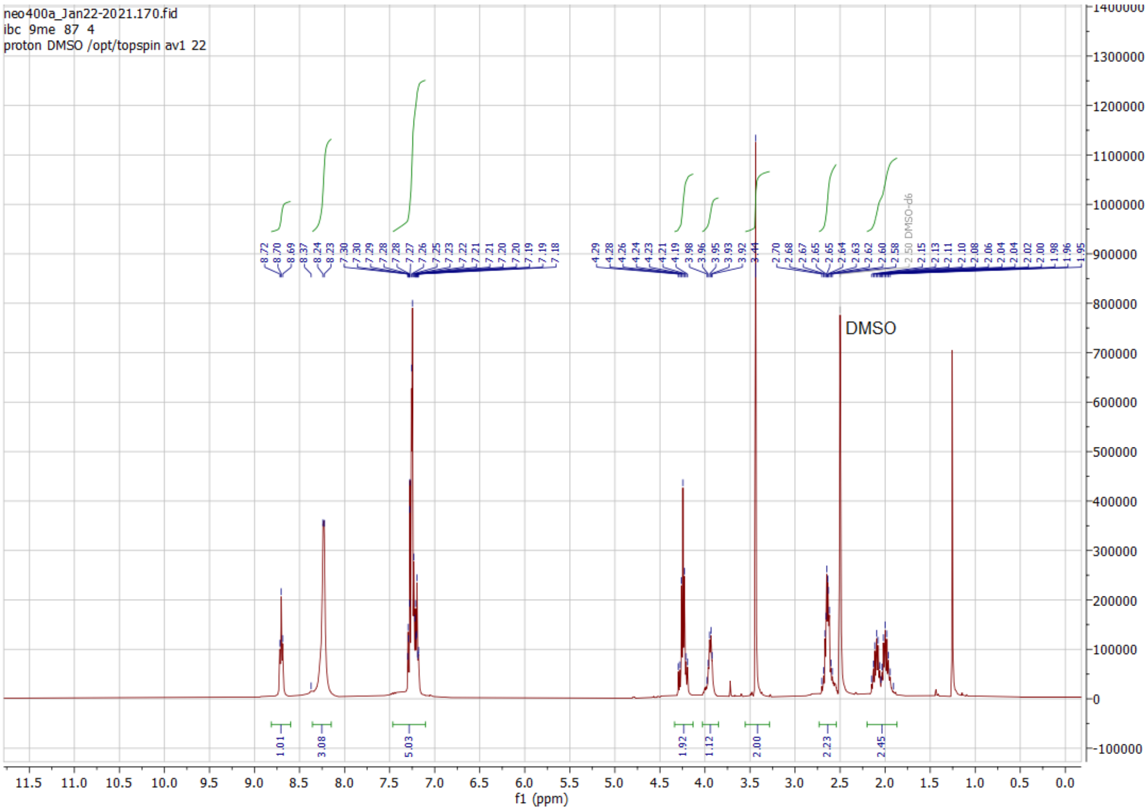

^{13}C NMR spectrum of HcP (**1**) TFA salt

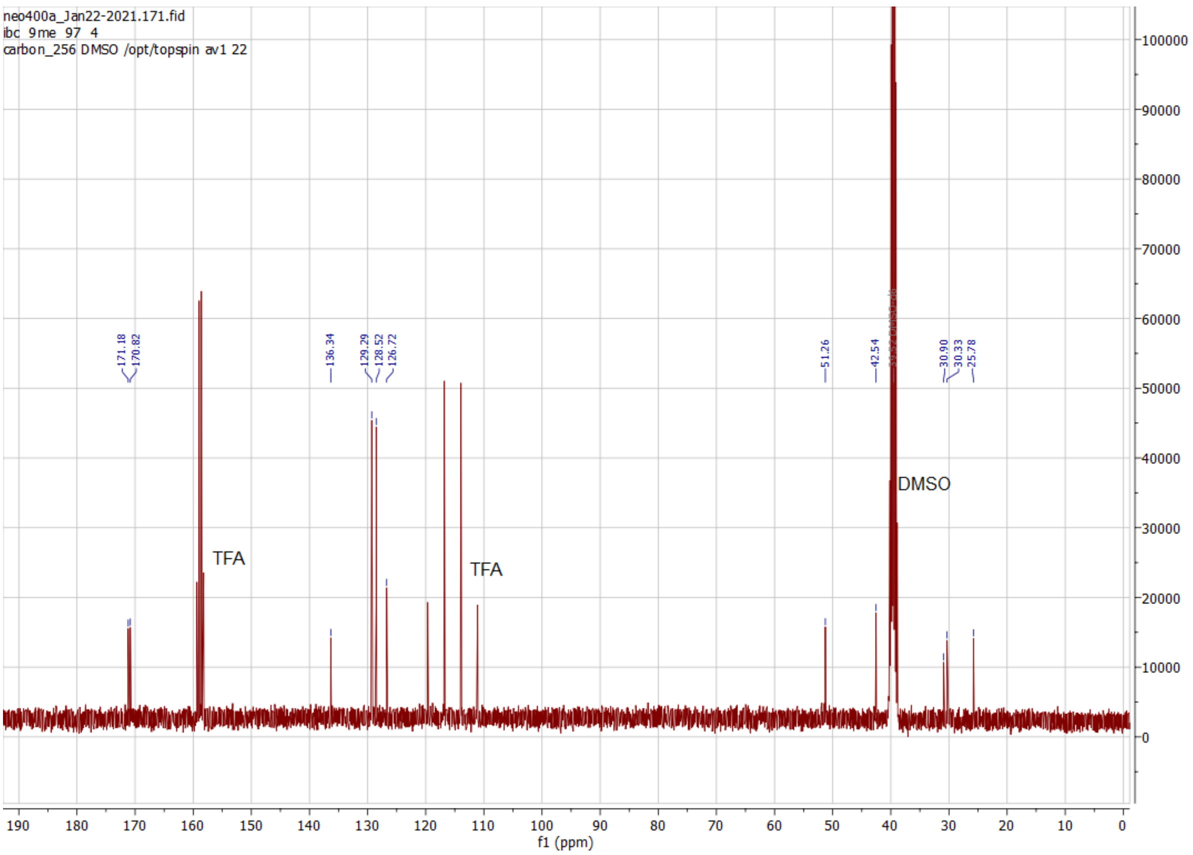

Supplement: Supplementary file 1 — Supplementary [file ANIE-60-15972-s001.pdf]
